# Common non-synonymous SNPs associated with breast cancer susceptibility: findings from the Breast Cancer Association Consortium

**DOI:** 10.1093/hmg/ddu311

**Published:** 2014-06-18

**Authors:** Roger L. Milne, Barbara Burwinkel, Kyriaki Michailidou, Jose-Ignacio Arias-Perez, M. Pilar Zamora, Primitiva Menéndez-Rodríguez, David Hardisson, Marta Mendiola, Anna González-Neira, Guillermo Pita, M. Rosario Alonso, Joe Dennis, Qin Wang, Manjeet K. Bolla, Anthony Swerdlow, Alan Ashworth, Nick Orr, Minouk Schoemaker, Yon-Dschun Ko, Hiltrud Brauch, Ute Hamann, The GENICA Network, Irene L. Andrulis, Julia A. Knight, Gord Glendon, Sandrine Tchatchou, kConFab Investigators, Australian Ovarian Cancer Study Group, Keitaro Matsuo, Hidemi Ito, Hiroji Iwata, Kazuo Tajima, Jingmei Li, Judith S. Brand, Hermann Brenner, Aida Karina Dieffenbach, Volker Arndt, Christa Stegmaier, Diether Lambrechts, Gilian Peuteman, Marie-Rose Christiaens, Ann Smeets, Anna Jakubowska, Jan Lubinski, Katarzyna Jaworska-Bieniek, Katazyna Durda, Mikael Hartman, Miao Hui, Wei Yen Lim, Ching Wan Chan, Federick Marme, Rongxi Yang, Peter Bugert, Annika Lindblom, Sara Margolin, Montserrat García-Closas, Stephen J. Chanock, Jolanta Lissowska, Jonine D. Figueroa, Stig E. Bojesen, Børge G. Nordestgaard, Henrik Flyger, Maartje J. Hooning, Mieke Kriege, Ans M.W. van den Ouweland, Linetta B. Koppert, Olivia Fletcher, Nichola Johnson, Isabel dos-Santos-Silva, Julian Peto, Wei Zheng, Sandra Deming-Halverson, Martha J. Shrubsole, Jirong Long, Jenny Chang-Claude, Anja Rudolph, Petra Seibold, Dieter Flesch-Janys, Robert Winqvist, Katri Pylkäs, Arja Jukkola-Vuorinen, Mervi Grip, Angela Cox, Simon S. Cross, Malcolm W.R. Reed, Marjanka K. Schmidt, Annegien Broeks, Sten Cornelissen, Linde Braaf, Daehee Kang, Ji-Yeob Choi, Sue K. Park, Dong-Young Noh, Jacques Simard, Martine Dumont, Mark S. Goldberg, France Labrèche, Peter A. Fasching, Alexander Hein, Arif B. Ekici, Matthias W. Beckmann, Paolo Radice, Paolo Peterlongo, Jacopo Azzollini, Monica Barile, Elinor Sawyer, Ian Tomlinson, Michael Kerin, Nicola Miller, John L. Hopper, Daniel F. Schmidt, Enes Makalic, Melissa C. Southey, Soo Hwang Teo, Cheng Har Yip, Kavitta Sivanandan, Wan-Ting Tay, Chen-Yang Shen, Chia-Ni Hsiung, Jyh-Cherng Yu, Ming-Feng Hou, Pascal Guénel, Therese Truong, Marie Sanchez, Claire Mulot, William Blot, Qiuyin Cai, Heli Nevanlinna, Taru A. Muranen, Kristiina Aittomäki, Carl Blomqvist, Anna H. Wu, Chiu-Chen Tseng, David Van Den Berg, Daniel O. Stram, Natalia Bogdanova, Thilo Dörk, Kenneth Muir, Artitaya Lophatananon, Sarah Stewart-Brown, Pornthep Siriwanarangsan, Arto Mannermaa, Vesa Kataja, Veli-Matti Kosma, Jaana M. Hartikainen, Xiao-Ou Shu, Wei Lu, Yu-Tang Gao, Ben Zhang, Fergus J. Couch, Amanda E. Toland, TNBCC, Drakoulis Yannoukakos, Suleeporn Sangrajrang, James McKay, Xianshu Wang, Janet E. Olson, Celine Vachon, Kristen Purrington, Gianluca Severi, Laura Baglietto, Christopher A. Haiman, Brian E. Henderson, Fredrick Schumacher, Loic Le Marchand, Peter Devilee, Robert A.E.M. Tollenaar, Caroline Seynaeve, Kamila Czene, Mikael Eriksson, Keith Humphreys, Hatef Darabi, Shahana Ahmed, Mitul Shah, Paul D.P. Pharoah, Per Hall, Graham G. Giles, Javier Benítez, Alison M. Dunning, Georgia Chenevix-Trench, Douglas F. Easton, Andrew Berchuck, Rosalind A. Eeles, Ali Amin Al Olama, Zsofia Kote-Jarai, Sara Benlloch, Antonis Antoniou, Lesley McGuffog, Ken Offit, Andrew Lee, Ed Dicks, Craig Luccarini, Daniel C. Tessier, Francois Bacot, Daniel Vincent, Sylvie LaBoissière, Frederic Robidoux, Sune F. Nielsen, Julie M. Cunningham, Sharon A. Windebank, Christopher A. Hilker, Jeffrey Meyer, Maggie Angelakos, Judi Maskiell, Ellen van der Schoot, Emiel Rutgers, Senno Verhoef, Frans Hogervorst, Prat Boonyawongviroj, Pornthep Siriwanarungsan, Michael Schrauder, Matthias Rübner, Sonja Oeser, Silke Landrith, Eileen Williams, Elaine Ryder-Mills, Kara Sargus, Niall McInerney, Gabrielle Colleran, Andrew Rowan, Angela Jones, Christof Sohn, Andeas Schneeweiß, Peter Bugert, Núria Álvarez, James Lacey, Sophia Wang, Huiyan Ma, Yani Lu, Dennis Deapen, Rich Pinder, Eunjung Lee, Fred Schumacher, Pam Horn-Ross, Peggy Reynolds, David Nelson, Hartwig Ziegler, Sonja Wolf, Volker Hermann, Wing-Yee Lo, Christina Justenhoven, Christian Baisch, Hans-Peter Fischer, Thomas Brüning, Beate Pesch, Sylvia Rabstein, Anne Lotz, Volker Harth, Tuomas Heikkinen, Irja Erkkilä, Kirsimari Aaltonen, Karl von Smitten, Natalia Antonenkova, Peter Hillemanns, Hans Christiansen, Eija Myöhänen, Helena Kemiläinen, Heather Thorne, Eveline Niedermayr, D Bowtell, G Chenevix-Trench, A deFazio, D Gertig, A Green, P Webb, A. Green, P. Parsons, N. Hayward, P. Webb, D. Whiteman, Annie Fung, June Yashiki, Gilian Peuteman, Dominiek Smeets, Thomas Van Brussel, Kathleen Corthouts, Nadia Obi, Judith Heinz, Sabine Behrens, Ursula Eilber, Muhabbet Celik, Til Olchers, Siranoush Manoukian, Bernard Peissel, Giulietta Scuvera, Daniela Zaffaroni, Bernardo Bonanni, Irene Feroce, Angela Maniscalco, Alessandra Rossi, Loris Bernard, Martine Tranchant, Marie-France Valois, Annie Turgeon, Lea Heguy, Phuah Sze Yee, Peter Kang, Kang In Nee, Shivaani Mariapun, Yoon Sook-Yee, Daphne Lee, Teh Yew Ching, Nur Aishah Mohd Taib, Meeri Otsukka, Kari Mononen, Teresa Selander, Nayana Weerasooriya, OFBCR staff, E. Krol-Warmerdam, J. Molenaar, J. Blom, Louise Brinton, Neonila Szeszenia-Dabrowska, Beata Peplonska, Witold Zatonski, Pei Chao, Michael Stagner, Petra Bos, Jannet Blom, Ellen Crepin, Anja Nieuwlaat, Annette Heemskerk, Sue Higham, Simon Cross, Helen Cramp, Dan Connley, Sabapathy Balasubramanian, Ian Brock, Craig Luccarini, Don Conroy, Caroline Baynes, Kimberley Chua

**Affiliations:** 1 Cancer Epidemiology Centre, Cancer Council Victoria, Melbourne, Australia,; 2 Centre for Epidemiology and Biostatistics, Melbourne School of Population and Global Health,; 3 Department of Pathology, The University of Melbourne, Melbourne, Australia,; 4 Human Cancer Genetics Programme,; 5 Human Genotyping-CEGEN Unit, Spanish National Cancer Research Centre (CNIO), Madrid, Spain,; 6 Department of Obstetrics and Gynecology,; 7 National Center for Tumor Diseases, University of Heidelberg, Heidelberg, Germany,; 8 Molecular Epidemiology Group,; 9 Division of Clinical Epidemiology and Aging Research,; 10 Division of Cancer Epidemiology, German Cancer Research Center (DKFZ), Heidelberg, Germany,; 11 Centre for Cancer Genetic Epidemiology, Department of Public Health and Primary Care,; 12 Centre for Cancer Genetic Epidemiology, Department of Oncology, University of Cambridge, Cambridge, UK,; 13 Servicio de Cirugía General y Especialidades, Hospital Monte Naranco, Oviedo, Spain,; 14 Servicio de Oncología Médica, Hospital Universitario La Paz, Madrid, Spain,; 15 Department of Pathology,Hospital Universitario La Paz, IdiPAZ (Hospital La Paz Institute for Health Research) Universidad Autonoma de Madrid, Madrid, Spain,; 16 Laboratory of Pathology and Oncology, Research Unit, Hospital Universitario La Paz, IdiPAZ, Madrid, Spain,; 17 Division of Genetics and Epidemiology, The Institute of Cancer Research, Sutton, UK,; 18 Division of Breast Cancer Research,; 19 Breakthrough Breast Cancer Research Centre, Division of Breast Cancer Research,; 20 Breakthrough Breast Cancer Research Centre,; 21 Division of Genetics and Epidemiology, The Institute of Cancer Research, London, UK,; 22 Department of Internal Medicine, Evangelische Kliniken Bonn gGmbH, Johanniter Krankenhaus, Bonn, Germany,; 23 Dr. Margarete Fischer-Bosch Institute of Clinical Pharmacology, Stuttgart, Germany,; 24 University of Tübingen, Tübingen, Germany,; 25 Molecular Genetics of Breast Cancer, Deutsches Krebsforschungszentrum (DKFZ), Heidelberg, Germany,; 26 Institute for Prevention and Occupational Medicine of the German Social Accident Insurance, Institute of the Ruhr-University Bochum (IPA), Bochum, Germany,; 27 Institute for Occupational Medicine and Maritime Medicine, University Medical Center Hamburg-Eppendorf, Hamburg, Germany,; 28 Institute of Pathology, Medical Faculty of the University of Bonn, Bonn, Germany,; 29 Lunenfeld-Tanenbaum Research Institute, Mount Sinai Hospital, Toronto, ON, Canada,; 30 Department of Molecular Genetics,; 31 Division of Epidemiology, Dalla Lana School of Public Health, University of Toronto, Toronto, ON, Canada,; 32 Ontario Cancer Genetics Network, Lunenfeld-Tanenbaum Research Institute, Toronto, ON, Canada,; 33 Peter MacCallum Cancer Centre, Melbourne, Australia,; 34 QIMR Berghofer Institute of Medical Research, Brisbane, Australia,; 35 Department of Preventive Medicine, Kyushu University Faculty of Medical Sciences, Fukuoka, Japan,; 36 Division of Epidemiology and Prevention, Aichi Cancer Center Research Institute, Nagoya, Japan,; 37 Department of Breast Oncology, Aichi Cancer Center Hospital, Nagoya, Japan,; 38 Department of Public Health & Occupational Medicine, Mie University Graduate School of Medicine, Tsu, Japan,; 39 Human Genetics Division, Genome Institute of Singapore, Singapore,; 40 Department of Medical Epidemiology and Biostatistics,; 41 Department of Molecular Medicine and Surgery,; 42 Department of Oncology and Pathology, Karolinska Institutet, Stockholm, Sweden,; 43 German Cancer Consortium (DKTK), Heidelberg, Germany,; 44 Saarland Cancer Registry, Saarbrücken, Germany,; 45 Vesalius Research Center (VRC), VIB, Leuven, Belgium,; 46 Multidisciplinary Breast Center, University Hospital Gasthuisberg, Leuven, Belgium,; 47 Department of Genetics and Pathology, Pomeranian Medical University, Szczecin, Poland,; 48 Saw Swee Hock School of Public Health, Department of Surgery, Yong Loo Lin School of Medicine,; 49 Saw Swee Hock School of Public Health, National University of Singapore and National University Health System, Singapore, Singapore,; 50 Department of Surgery, National University Health System, Singapore, Singapore,; 51 Institute of Transfusion Medicine and Immunology, Medical Faculty Mannheim, Heidelberg University, Mannheim, Germany,; 52 Division of Cancer Epidemiology and Genetics, National Cancer Institute, Rockville, MD, USA,; 53 Department of Cancer Epidemiology and Prevention, M. Sklodowska-Curie Memorial Cancer Center & Institute of Oncology, Warsaw, Poland,; 54 Copenhagen General Population Study, Herlev Hospital,; 55 Department of Clinical Biochemistry, Herlev Hospital,; 56 Department of Breast Surgery, Herlev Hospital, Copenhagen University Hospital, Copenhagen, Denmark,; 57 Faculty of Health and Medical Sciences, University of Copenhagen, Copenhagen, Denmark,; 58 Department of Medical Oncology,; 59 Department of Surgical Oncology, Family Cancer Clinic, Erasmus MC Cancer Institute, Rotterdam, The Netherlands,; 60 Department of Clinical Genetics, Erasmus University Medical Center, Rotterdam, The Netherlands,; 61 London School of Hygiene and Tropical Medicine, London, UK,; 62 Division of Epidemiology, Department of Medicine, Vanderbilt University Medical Center, Nashville, TN, USA,; 63 Institute for Medical Biometrics and Epidemiology,; 64 Department of Cancer Epidemiology/Clinical Cancer Registry, University Clinic Hamburg-Eppendorf, Hamburg, Germany,; 65 Laboratory of Cancer Genetics and Tumor Biology, Department of Clinical Chemistry and Biocenter Oulu, University of Oulu, Northern Finland Laboratory Centre NordLab, Oulu, Finland,; 66 Department of Oncology,; 67 Department of Surgery, Oulu University Hospital, University of Oulu, Oulu, Finland,; 68 CRUK/YCR Sheffield Cancer Research Centre, Department of Oncology,; 69 Academic Unit of Pathology, Department of Neuroscience, University of Sheffield, Sheffield, South Yorkshire, UK,; 70 Netherlands Cancer Institute, Antoni van Leeuwenhoek Hospital, Amsterdam, The Netherlands,; 71 Department of Preventive Medicine,; 72 Department of Surgery, Seoul National University College of Medicine, Seoul, Korea,; 73 Department of Biomedical Sciences, Seoul National University Graduate School, Seoul, Korea,; 74 Cancer Research Institute, Seoul National University, Seoul, Korea,; 75 Genomics Center, Centre Hospitalier Universitaire de Québec Research Center and Laval University, QC, Canada,; 76 Department of Medicine, McGill University, Montreal, QC, Canada,; 77 Division of Clinical Epidemiology, McGill University Health Centre, Royal Victoria Hospital, Montreal, QC, Canada,; 78 Département de médecine sociale et préventive, Département de santé environnementale et santé au travail, Université de Montréal, Montreal, QC, Canada,; 79 University Breast Center Franconia, Department of Gynecology and Obstetrics,; 80 Institute of Human Genetics, University Hospital Erlangen, Friedrich-Alexander University Erlangen-Nuremberg, Comprehensive Cancer Center Erlangen-EMN, Erlangen, Germany,; 81 David Geffen School of Medicine, Department of Medicine Division of Hematology and Oncology, University of California at Los Angeles, CA, USA,; 82 Unit of Molecular Bases of Genetic Risk and Genetic Testing, Department of Preventive and Predictive Medicine,; 83 Unit of Medical Genetics, Department of Preventive and Predictive Medicine, Fondazione IRCCS Istituto Nazionale dei Tumori (INT), Milan, Italy,; 84 IFOM, Fondazione Istituto FIRC di Oncologia Molecolare, Milan, Italy,; 85 Division of Cancer Prevention and Genetics, Istituto Europeo di Oncologia (IEO), Milan, Italy,; 86 Division of Cancer Studies, NIHR Comprehensive Biomedical Research Centre, Guy's & St. Thomas’ NHS Foundation Trust in partnership with King's College London, London, UK,; 87 Wellcome Trust Centre for Human Genetics,; 88 Oxford Biomedical Research Centre, University of Oxford, Oxford, UK,; 89 School of Medicine, Clinical Science Institute, National University of Ireland, Galway, Ireland,; 90 Cancer Research Initiatives Foundation, Sime Darby Medical Centre, Subang Jaya, Malaysia,; 91 Breast Cancer Research Unit, University Malaya Cancer Research Institute, University Malaya Medical Centre, Kuala Lumpur, Malaysia,; 92 Singapore Eye Research Institute, National University of Singapore, Singapore, Singapore,; 93 Institute of Biomedical Sciences, Academia Sinica, Taipei, Taiwan,; 94 College of Public Health, China Medical University, Taichong, Taiwan,; 95 Tri-Service General Hospital, Taipei, Taiwan,; 96 Cancer Center,; 97 Department of Surgery, Kaohsiung Medical University Chung-Ho Memorial Hospital, Kaohsiung, Taiwan,; 98 Inserm (National Institute of Health and Medical Research), CESP (Center for Research in Epidemiology and Population Health), U1018, Environmental Epidemiology of Cancer, Villejuif, France,; 99 University Paris-Sud, UMRS 1018, Villejuif, France,; 100 Inserm (National Institute of Health and Medical Research), U775, Paris, France,; 101 Centre de Ressources Biologiques EPIGENETEC, Paris, France,; 102 Department of Medicine, Vanderbilt University, Nashville, TN, USA,; 103 Department of Obstetrics and Gynecology, University of Helsinki and Helsinki University Central Hospital, Helsinki, Finland,; 104 Department of Clinical Genetics,; 105 Department of Oncology, Helsinki University Central Hospital, Helsinki, Finland,; 106 Department of Preventive Medicine, Keck School of Medicine, University of Southern California, Los Angeles, CA, USA,; 107 Department of Obstetrics and Gynaecology,; 108 Department of Radiation Oncology, Hannover Medical School, Hannover, Germany,; 109 Institute of Population Health, University of Manchester, Manchester, UK,; 110 Division of Health Sciences, Warwick Medical School, Coventry, UK,; 111 Ministry of Public Health, Thailand,; 112 School of Medicine, Institute of Clinical Medicine, Pathology and Forensic Medicine,; 113 Biocenter Kuopio,; 114 School of Medicine, Institute of Clinical Medicine, Oncology, University of Eastern Finland, Kuopio, Finland,; 115 Department of Clinical Pathology,; 116 Cancer Center, Kuopio University Hospital, Kuopio, Finland,; 117 Shanghai Center for Disease Control and Prevention, Shanghai, China,; 118 Shanghai Cancer Institute, Shanghai, China,; 119 Department of Laboratory Medicine and Pathology,; 120 Department of Health Sciences Research,; 121 Mayo Clinic, Rochester, MN, USA,; 122 Department of Molecular Virology, Immunology and Medical Genetics, Comprehensive Cancer Center, The Ohio State University, Columbus, OH, USA,; 123 Molecular Diagnostics Laboratory, INRASTES, National Centre for Scientific Research ‘Demokritos’, Athens, Greece,; 124 National Cancer Institute, Bangkok, Thailand,; 125 Genetic Susceptibility Group, International Agency for Research on Cancer, Lyon, France,; 126 University of Hawaii Cancer Center, Honolulu, HI, USA,; 127 Department of Human Genetics and; 128 Department of Surgical Oncology, Leiden University Medical Center, Leiden, The Netherlands

## Abstract

Candidate variant association studies have been largely unsuccessful in identifying common breast cancer susceptibility variants, although most studies have been underpowered to detect associations of a realistic magnitude. We assessed 41 common non-synonymous single-nucleotide polymorphisms (nsSNPs) for which evidence of association with breast cancer risk had been previously reported. Case-control data were combined from 38 studies of white European women (46 450 cases and 42 600 controls) and analyzed using unconditional logistic regression. Strong evidence of association was observed for three nsSNPs: *ATXN7-*K264R at 3p21 [rs1053338, per allele OR = 1.07, 95% confidence interval (CI) = 1.04–1.10, *P* = 2.9 × 10^−6^], *AKAP9-*M463I at 7q21 (rs6964587, OR = 1.05, 95% CI = 1.03–1.07, *P* = 1.7 × 10^−6^) and *NEK10-*L513S at 3p24 (rs10510592, OR = 1.10, 95% CI = 1.07–1.12, *P* = 5.1 × 10^−17^). The first two associations reached genome-wide statistical significance in a combined analysis of available data, including independent data from nine genome-wide association studies (GWASs): for *ATXN7-*K264R, OR = 1.07 (95% CI = 1.05–1.10, *P* = 1.0 × 10^−8^); for *AKAP9-*M463I, OR = 1.05 (95% CI = 1.04–1.07, *P* = 2.0 × 10^−10^). Further analysis of other common variants in these two regions suggested that intronic SNPs nearby are more strongly associated with disease risk. We have thus identified a novel susceptibility locus at 3p21, and confirmed previous suggestive evidence that rs6964587 at 7q21 is associated with risk. The third locus, rs10510592, is located in an established breast cancer susceptibility region; the association was substantially attenuated after adjustment for the known GWAS hit. Thus, each of the associated nsSNPs is likely to be a marker for another, non-coding, variant causally related to breast cancer risk. Further fine-mapping and functional studies are required to identify the underlying risk-modifying variants and the genes through which they act.

## INTRODUCTION

Few common non-synonymous genetic variants have been implicated in breast cancer susceptibility. Earlier candidate–gene association studies focused heavily on such variants but generally failed to produce robust findings (1). Agnostic approaches using genome-wide panels of single-nucleotide polymorphisms (SNPs) have been much more successful, having identified >70 common breast cancer susceptibility loci to date (2–21). No missense variants have been clearly shown to explain these observed associations with marker SNPs. The fact that the effect sizes detected by these large-scale studies were relatively small [for the vast majority, the associated odds ratio (OR) was <1.20] suggests that most, if not all, of the earlier candidate-gene studies were underpowered to detect associations of a realistic magnitude.

The Wellcome Trust Case-Control Consortium (WTCCC) previously conducted an association study of 14 436 non-synonymous SNPs (nsSNPs) across the genome, using a custom array genotyped in 1053 breast cancer cases and 1500 controls (22). No clear associations were identified. However, no replication stage was carried out and the study had <15% power to detect a per-allele OR of 1.20 for even the most common variants at a Bonferroni-corrected nominal significance threshold of 3.5 × 10^−6^. One of the SNPs on the array has previously been studied by Breast Cancer Association Consortium (BCAC); we found evidence that *AKAP9*-M463I (rs6964587) was associated with breast cancer risk, with a recessive model appearing to be the best fit, although evidence of association (*P* = 0.001) did not reach genome-wide statistical significance (23).

We aimed to assess the most promising association signals from the WTCCC study in a much larger BCAC case–control study that formed part of the Collaborative Oncological Gene-Environment Study (COGS). COGS is a multi-consortium project that seeks to identify common variants contributing to susceptibility to breast, ovarian and prostate cancer (http://www.nature.com/icogs/primer/cogs-project-and-design-of-the-icogs-array/). It is based on genotyping case–control samples using a custom iSelect SNP genotyping array (iCOGS). The principal criterion for inclusion of SNPs on this array by BCAC was statistical evidence of association from a combined analysis of nine genome-wide association studies (GWASs); the analysis of these SNPs selected from GWAS, identifying >40 novel breast cancer susceptibility loci (2–4), has been completed. We also included on the iCOGS array, and successfully genotyped, 41 nsSNPs from the WTCCC study, including rs6964587, for which the strongest evidence of association had been observed. In the present analysis, we attempted to replicate these associations using the BCAC component of COGS, comprising 53 835 female breast cancer cases and 50 156 controls (Table 1).

**Table 1.** BCAC studies contributing cases and controls to COGS

Study	Country	Controls	Cases	ER+	ER−
European women					
Australian Breast Cancer Family Study^a^ (ABCFS)	Australia	551	790	456	261
Amsterdam Breast Cancer Study (ABCS)	Netherlands	1429	1325	420	153
Bavarian Breast Cancer Cases and Controls (BBCC)	Germany	458	564	460	83
British Breast Cancer Study (BBCS)	UK	1397	1554	507	114
Breast Cancer In Galway Genetic Study (BIGGS)	Ireland	719	836	495	154
Breast Cancer Study of the University Clinic Heidelberg (BSUCH)	Germany	954	852	499	154
CECILE Breast Cancer Study (CECILE)	France	999	1019	797	144
Copenhagen General Population Study (CGPS)	Denmark	4086	2901	1919	357
Spanish National Cancer Research Centre Breast Cancer Study (CNIO-BCS)	Spain	876	902	242	88
California Teachers Study (CTS)	USA	71	68	0	17
ESTHER Breast Cancer Study (ESTHER)	Germany	502	478	304	98
Gene Environment Interaction and Breast Cancer in Germany (GENICA)	Germany	427	465	328	119
Helsinki Breast Cancer Study (HEBCS)	Finland	1234	1664	1295	237
Hannover-Minsk Breast Cancer Study (HMBCS)	Belarus	130	690	37	0
Karolinska Breast Cancer Study (KARBAC)	Sweden	662	722	338	63
Kuopio Breast Cancer Project (KBCP)	Finland	251	445	304	97
kConFab/Australian Ovarian Cancer Study (kConFab/AOCS)	Australia	897	613	162	59
Leuven Multidisciplinary Breast Centre (LMBC)	Belgium	1388	2671	2071	379
Mammary Carcinoma Risk Factor Investigation (MARIE)	Germany	1778	1818	1349	399
Milan Breast Cancer Study Group (MBCSG)	Italy	400	488	149	42
Mayo Clinic Breast Cancer Study (MCBCS)	USA	1931	1862	1486	295
Melbourne Collaborative Cohort Study (MCCS)	Australia	511	614	352	119
Multi-ethnic Cohort (MEC)	USA	741	731	415	87
Montreal Gene-Environment Breast Cancer Study (MTLGEBCS)	Canada	436	489	421	64
Norwegian Breast Cancer Study (NBCS)	Norway	70	22	0	22
Oulu Breast Cancer Study (OBCS)	Finland	414	507	407	100
Ontario Familial Breast Cancer Registry^b^ (OFBCR)	Canada	511	1175	630	268
Leiden University Medical Centre Breast Cancer Study (ORIGO)	Netherlands	327	357	211	70
NCI Polish Breast Cancer Study (PBCS)	Poland	424	519	519	0
Karolinska Mammography Project for Risk Prediction of Breast Cancer (pKARMA)	Sweden	5,537	5434	3672	702
Rotterdam Breast Cancer Study (RBCS)	Netherlands	699	664	368	131
Singapore and Sweden Breast Cancer Study (SASBAC)	Sweden	1378	1163	663	144
Sheffield Breast Cancer Study (SBCS)	UK	848	843	377	105
Study of Epidemiology and Risk factors in Cancer Heredity (SEARCH)	UK	8069	9347	5160	1181
Städtisches Klinikum Karlsruhe Deutsches Krebsforschungszentrum Study (SKKDKFZS)	Germany	29	136	0	136
Szczecin Breast Cancer Study (SZBCS)	Poland	315	365	165	60
Triple Negative Breast Cancer Consortium Study (TNBCC)	Various	542	881	0	881
UK Breakthrough Generations Study (UKBGS)	UK	470	476	96	22
Asian women					
Asian Cancer Project (ACP)	Thailand	636	423	92	53
Hospital-based Epidemiologic Research Program at Aichi Cancer Center (HERPACC)	Japan	1376	694	395	139
Los Angeles County Asian-American Breast Cancer Case-Control (LAABC)	USA	990	812	528	138
Malaysian Breast Cancer Genetic Study (MYBRCA)	Malaysia	610	770	422	291
Shanghai Breast Cancer Genetic Study (SBCGS)	China	892	848	510	276
Seoul Breast Cancer Study (SEBCS)	South Korea	1129	1162	657	375
Singapore Breast Cancer Cohort (SGBCC)	Singapore	502	533	272	108
IARC-Thai Breast Cancer (TBCS)	Thailand	253	138	26	26
Taiwanese Breast Cancer Study (TWBCS)	Taiwan	236	889	460	204
African-American women					
Southern Community Cohort Study (SCCS)	USA	680	679	0	0
Nashville Breast Health Study (NBHS)	USA	252	437	199	222
Total		50 156	53 835	30 635	9120

BCAC, Breast Cancer Association Consortium; COGS, Collaborative Oncological Gene-Environment Study; ER+, estrogen receptor-positive cases; ER−, estrogen receptor-negative cases.

^a^Australian site of the Breast Cancer Family Registry.

^b^Ontario site of the Breast Cancer Family Registry.

## RESULTS

After quality control (QC), all genotyped SNPs in the present analysis had overall call rates >95% and duplicate and HapMap sample concordance >98%. No evidence of departure from Hardy–Weinberg equilibrium was observed in controls overall (*P* ≥ 0.11 for Europeans), and no strong evidence was seen in controls from any single study (*P* ≥ 2.3 × 10^−4^). Results from analysis of main effects for Europeans (46 450 cases and 42 600 controls) are summarized in Table 2. No notable between-study heterogeneity was observed for any SNP (*I*^2^ ≤ 33%). Nominally statistically significant associations (*P* < 0.05) were observed for seven SNPs; however, for four of these the evidence of association was weak (*P* ≥ 0.012) and compatible with chance association, given the number of SNPs considered. Stronger evidence of association was observed for three SNPs: rs10510592 (L513S) in *NEK10* [per-allele odds ratio (OR) = 1.10, 95% CI = 1.07–1.12; *P* = 5.1 × 10^−17^], rs6964587 (M463I) in *AKAP9* (per-allele OR = 1.05; 95% CI = 1.03–1.07; *P* = 1.7 × 10^−6^) and rs1053338 (K264R) in *ATXN7* (per-allele OR = 1.07; 95% CI = 1.04–1.10; *P* = 2.9 × 10^−6^). Subsequent analyses were focused on these three variants (see Supplementary Material, figure).

**Table 2.** Summary results from COGS-BCAC for European women

Original SNP (nsC)	Gene	Surrogate SNP^a^	Alleles^b^	MAF	pHWE	OR (95% CI) *P*-value^^c^^			P-het^d^	*I*^2^ (%)^d^
						Aa	aa	Per-a-allele		
rs10415312 (E171K)	*OR7C1*		AG	0.09	0.36	0.98 (0.95, 1.02) 0.31	1.02 (0.87, 1.17) 0.81	0.99 (0.96, 1.02) 0.43	0.38	5.05
rs10494217 (H50N)	*TBX15*		CA	0.19	0.28	1.00 (0.97, 1.03) 0.94	0.98 (0.91, 1.05) 0.60	1.00 (0.97, 1.02) 0.81	0.06	27.7
rs10510592 (L513S)	*NEK10*		AG	0.25	0.26	1.11 (1.08, 1.14) 1.4 × 10^−12^	1.18 (1.12, 1.25) 1.5 × 10^−9^	1.10 (1.07, 1.12) 5.1 × 10^−17^	0.53	0
rs1053338 (K264R)	*ATXN7*		AG	0.13	0.53	1.07 (1.03, 1.10) 5.6 × 10^−5^	1.14 (1.04, 1.26) 0.0073	1.07 (1.04, 1.10) 2.9 × 10^−6^	0.23	13.7
rs11078738 (L621P)	*PFAS*		GA	0.24	0.41	1.01 (0.98, 1.03) 0.73	0.96 (0.90, 1.02) 0.15	0.99 (0.97, 1.01) 0.50	0.27	11.1
rs12051468 (S105G)	*CRISPLD2*		AG	0.43	0.39	1.02 (0.99, 1.05) 0.25	1.01 (0.97, 1.05) 0.66	1.01 (0.99, 1.03) 0.53	0.34	7.10
rs12256835 (H1759Q)	*C10orf112*		AC	0.18	0.25	1.01 (0.98, 1.04) 0.71	1.05 (0.97, 1.13) 0.25	1.01 (0.99, 1.04) 0.35	0.12	21.8
rs1265096 (E34K)	*PSORS1C1*		GA	0.09	0.39	1.02 (0.98, 1.06) 0.26	0.86 (0.74, 1.01) 0.061	1.00 (0.97, 1.04) 0.80	0.84	0
rs12894584 (intronic)	*NAA30*		GA	0.29	0.31	1.00 (0.97, 1.03) 0.96	0.98 (0.93, 1.03) 0.43	0.99 (0.97, 1.02) 0.60	0.65	0
rs13096522 (non-coding)	*ARL6*		TA	0.20	0.31	1.02 (0.99, 1.05) 0.13	0.99 (0.92, 1.06) 0.79	1.01 (0.99, 1.04) 0.34	0.77	0
rs1801197 (L447P)	*CALCR*	rs2023778, *r*^2^ = 1.0	AG	0.24	0.30	1.02 (0.99, 1.04) 0.27	1.02 (0.96, 1.08) 0.53	1.01 (0.99, 1.04) 0.26	0.85	0
rs2107732 (V53I)	*CCM2*		GA	0.09	0.48	0.99 (0.96, 1.03) 0.64	0.98 (0.84, 1.14) 0.80	0.99 (0.96, 1.02) 0.61	0.63	0
rs2230018 (T726K)	*KDM6A*		CA	0.12	0.43	0.98 (0.95, 1.01) 0.23	1.07 (0.96, 1.19) 0.23	0.99 (0.96, 1.02) 0.65	0.03	33.1
rs2272955 (M96T)	*WFDC8*		AG	0.05	0.31	1.02 (0.98, 1.07) 0.36	0.82 (0.64, 1.05) 0.12	1.01 (0.97, 1.05) 0.72	0.34	7.30
rs2282542 (V1365M)	*CEP192*		GA	0.12	0.42	0.97 (0.94, 1.01) 0.12	0.90 (0.80, 1.00) 0.050	0.97 (0.94, 1.00) 0.025	0.07	26.7
rs2285374^^e^^ (K889R)	*VPS11*		AG	0.39	0.32	0.99 (0.96, 1.02) 0.52	0.99 (0.95, 1.03) 0.67	0.99 (0.98, 1.01) 0.58	0.60	0
rs2286587 (R110H)	*MXRA7*		AG	0.39	0.50	0.97 (0.95, 1.00) 0.083	0.97 (0.94, 1.02) 0.29	0.99 (0.97, 1.01) 0.15	0.08	25.7
rs2291533 (Q253H)	*NIF3L1BP1*	rs7614311, *r*^2^ = 0.94	AC	0.19	0.34	1.03 (1.00, 1.06) 0.041	1.05 (0.98, 1.13) 0.14	1.03 (1.00, 1.05) 0.018	0.31	9.30
rs2298083 (V854I)	*SMG7*		GA	0.11	0.17	0.99 (0.95, 1.02) 0.39	1.02 (0.90, 1.15) 0.78	0.99 (0.96, 1.02) 0.54	0.71	0
rs2735018 (intronic)	*HLA-G*		GC	0.10	0.32	0.97 (0.94, 1.01) 0.13	0.94 (0.82, 1.07) 0.36	0.97 (0.94, 1.00) 0.086	0.38	5.30
rs2822558 (S199N)	*ABCC13*		GA	0.15	0.27	1.01 (0.98, 1.04) 0.52	1.01 (0.92, 1.10) 0.89	1.01 (0.98, 1.03) 0.56	0.60	0
rs2853699 (A27G)	*CCR8*	rs12107527, *r*^2^ = 1.0	GA	0.30	0.37	1.01 (0.98, 1.04) 0.64	1.01 (0.97, 1.06) 0.60	1.01 (0.99, 1.03) 0.53	0.26	11.8
rs2856705 (non-coding)	*HLA-DQA2*		GA	0.09	0.24	1.00 (0.96, 1.03) 0.87	1.07 (0.93, 1.22) 0.35	1.00 (0.97, 1.04) 0.79	0.07	26.5
rs2879097 (R79C)	*CISD3*		GA	0.22	0.50	1.00 (0.97, 1.03) 0.83	0.97 (0.91, 1.04) 0.38	0.99 (0.97, 1.01) 0.49	0.20	15.7
rs315675 (L396H)	*ZCCHC4*	rs13149511, *r*^2^ = 1.0	AG	0.11	0.37	1.00 (0.97, 1.03) 0.96	0.97 (0.85, 1.10) 0.61	1.00 (0.97, 1.03) 0.79	0.99	0
rs365990 (V1101A)	*MYH6*		AG	0.35	0.20	1.04 (1.01, 1.07) 0.014	1.04 (1.00, 1.09) 0.052	1.03 (1.01, 1.05) 0.012	0.13	20.4
rs3742801 (E368K)	*ABCD4*		GA	0.36	0.21	1.00 (0.97, 1.02) 0.76	1.02 (0.98, 1.06) 0.37	1.01 (0.99, 1.03) 0.57	0.10	23.2
rs3815768 (A298T)	*ELL2*		GA	0.26	0.38	1.00 (0.97, 1.03) 0.90	1.04 (0.98, 1.10) 0.16	1.01 (0.99, 1.03) 0.40	0.47	0
rs3873283 (non-coding)	*HCG9*	rs9260734, *r*^2^ = 1.0	GA	0.15	0.28	0.99 (0.96, 1.02) 0.51	0.95 (0.87, 1.04) 0.24	0.98 (0.96, 1.01) 0.25	0.54	0
rs3891175 (non-coding)	*HLA-DQB1*		GA	0.21	0.32	0.99 (0.96, 1.02) 0.64	0.97 (0.91, 1.03) 0.30	0.99 (0.97, 1.01) 0.34	0.22	14.2
rs3997854 (non-coding)	*HLA-DQA2*		AC	0.13	0.31	0.98 (0.95, 1.02) 0.33	0.93 (0.84, 1.03) 0.18	0.98 (0.95, 1.01) 0.14	0.73	0
rs4128458 (K323E)	*LAD1*		AG	0.50	0.27	0.99 (0.96, 1.03) 0.75	0.97 (0.94, 1.01) 0.18	0.99 (0.97, 1.01) 0.18	0.10	23.7
rs4986790 (D299G)	*TLR4*		AG	0.06	0.41	0.98 (0.94, 1.02) 0.38	0.96 (0.77, 1.20) 0.73	0.98 (0.94, 1.02) 0.35	0.40	4.12
rs5744751 (A252V)	*POLE*		GA	0.11	0.29	1.01 (0.98, 1.04) 0.57	1.02 (0.91, 1.15) 0.75	1.01 (0.98, 1.04) 0.52	0.87	0
rs6032538 (H36D)	*WFDC3*	rs399672, *r*^2^ = 1.0	AG	0.28	0.11	1.00 (0.97, 1.03) 0.82	0.98 (0.93, 1.03) 0.49	0.99 (0.97, 1.01) 0.55	0.11	22.0
rs6964587 (M463I)	*AKAP9*		GT	0.39	0.29	1.04 (1.01, 1.07) 0.0098	1.11 (1.06, 1.15) 1.6 × 10^−6^	1.05 (1.03, 1.07) 1.7 × 10^−6^	0.18	16.7
rs7158731 (L118P)	*ZNF839*		AG	0.18	0.33	1.00 (0.97, 1.03) 0.89	1.01 (0.94, 1.10) 0.71	1.00 (0.98, 1.03) 0.75	0.16	18.7
rs7454108 (non-coding)	*HLA-DQA2*		AG	0.11	0.26	0.96 (0.93, 1.00) 0.034	0.98 (0.87, 1.10) 0.73	0.97 (0.94, 1.00) 0.047	0.71	0
rs7863265 (F10L)	*STRBP*		GC	0.34	0.40	1.01 (0.98, 1.04) 0.48	1.00 (0.96, 1.05) 0.98	1.00 (0.98, 1.02) 0.75	0.43	2.42
rs8059973 (intronic)	*MGC3101*		GA	0.16	0.18	1.00 (0.97, 1.03) 0.83	1.00 (0.92, 1.08) 0.92	1.00 (0.98, 1.03) 0.91	0.55	0
rs9891699 (P19S)	*PFAS*		AG	0.19	0.48	1.01 (0.98, 1.04) 0.59	0.99 (0.92, 1.07) 0.87	1.00 (0.98, 1.03) 0.75	0.61	0

COGS, Collaborative Oncological Gene-environment Study; BCAC, Breast Cancer Association Consortium; nsC, non-synonymous amino acid change; MAF, minor allele frequency for controls; pHWE, *P*-value for compliance with Hardy–Weinberg equilibrium for controls; OR, odds ratio, where A is the common allele, a is the rare allele and both Aa and aa are compared with AA genotypes; CI, confidence interval; P-het, *P*-value for between-study homogeneity.

^a^SNP genotyped as a surrogate for the original SNP when the latter failed on design; *r*^2^ value given is that for LD between the surrogate and the original SNP; results in columns to the right are for the surrogate SNP.

^b^Minor allele listed second.

^c^Based on the Wald statistic for the genotype-specific estimates; based on the likelihood ratio test for the per-allele estimate.

^d^Applying the per-allele (log-additive) model.

^e^rs2285374 has been merged into rs15818.

SNP rs10510592 (L513S) in *NEK10* is located 83 kb from a known breast cancer susceptibility GWAS hit, rs4973768 (9), which was also genotyped on iCOGS; the two SNPs are in modest linkage disequilibrium (LD; *r*^2^ = 0.36). The evidence of association using the same dataset was stronger for rs4973768 (*P* = 3.0 × 10^−22^). A multivariate analysis including both SNPs resulted in substantial attenuation in the OR for rs10510592 (per-allele OR = 1.05, 95% CI = 1.02–1.07, *P* = 0.0010), while the evidence of association for rs4973768 remained strong (*P* = 1.0 × 10^−8^). The variant rs10510592 was included on iCOGS, both as part of the present study and as part of a fine-mapping study of 899 SNPs in an 881 kb region of 3p24. More detailed multivariate analyses of these fine-mapping SNPs, complemented by functional analysis, will be required to pinpoint the underlying causal variant(s).

**Figure 1.**
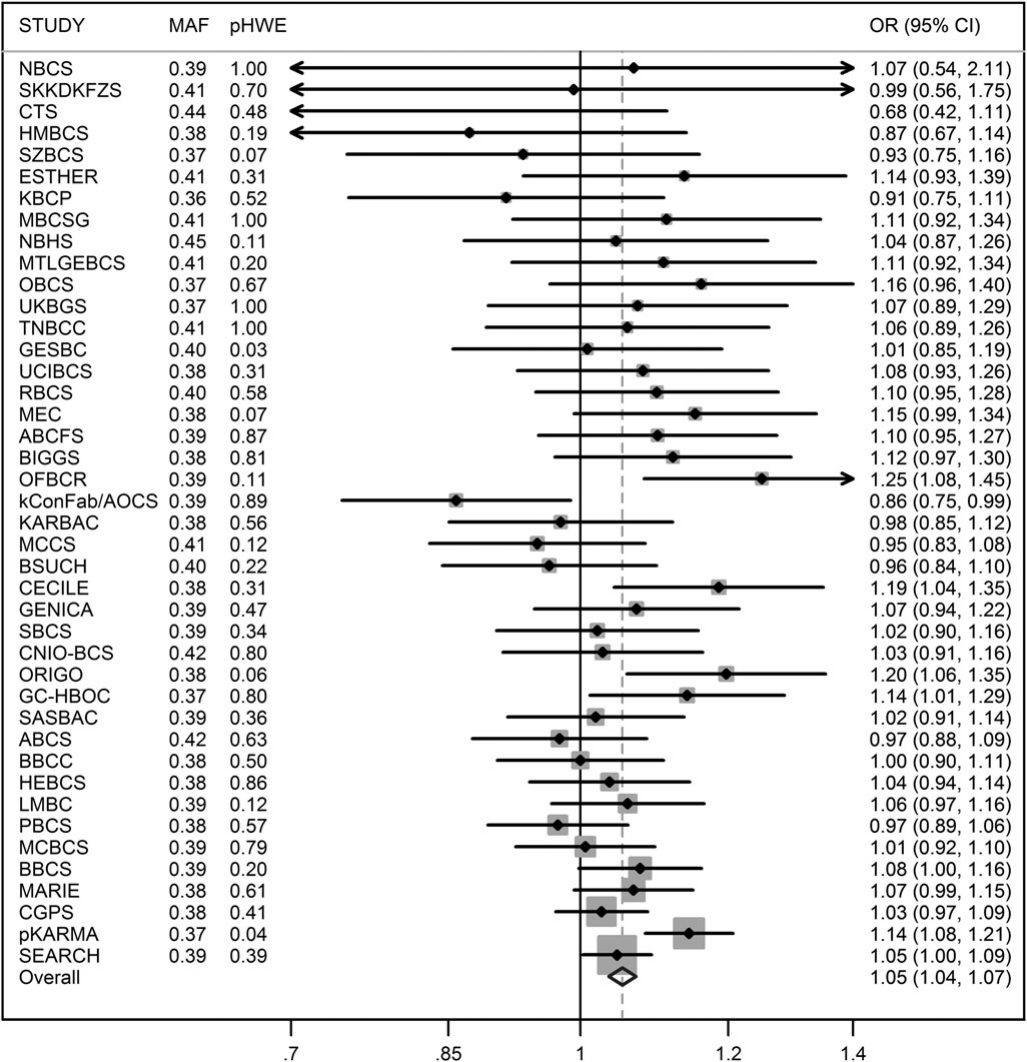
Per-allele OR estimates for *AKAP9*-M463I (rs6964587) for European women by study, based on published data and new data from the Breast Cancer Association Consoritum. MAF, minor allele frequency; pHWE, *P*-value for departure from Hardy–Weinberg equilibrium; CI, confidence interval.

The nsSNP in *AKAP9*, rs6964587, had been previously studied by the BCAC (23,24). The dataset used in the previous analysis overlapped partially with the present study (14 423 cases and 12 785 controls were in both datasets). Table 3 presents results from both analyses after removing overlapping samples from the latter. In the present study, we observed strong independent evidence of replication of the reported association (*P* = 9.2 × 10^−7^). After combining published and new data from European women (55 445 cases and 62 668 controls), the per-T-allele OR estimate was 1.05 (95% CI = 1.04–1.07, *P* = 2.5 × 10^−9^) and the OR relative to the GG genotype was 1.04 (95% CI = 1.01–1.07, *P* = 0.0034) for GT and 1.12 (95% CI = 1.08–1.16, *P* = 1.1 × 10^−9^) for TT. The per-allele OR estimates and 95% CI to two decimal places were unchanged when analyses were repeated excluding 3734 cases with carcinoma *in situ* or unknown invasiveness (*P* = 3.7 × 10^−9^). The above analyses were adjusted only for study as principal components could not be determined for published data; however, when adjustment was made for principal components for the iCOGS data alone (setting the principal components to zero for other samples), the results were similar (per-T-allele OR = 1.05, 95% CI = 1.03–1.07, *P* = 1.3 × 10^−8^). All subsequent analyses for this SNP included published and new data, unless otherwise specified. The genotype-specific ORs were consistent with a log-additive (per-allele) model; a recessive model as previously proposed could be rejected (OR = 1.05, 95% CI = 1.03–1.06, *P* = 8.4 × 10^−8^; *P* = 0.0034 compared with a two-parameter model). No notable between-study heterogeneity was observed (*I*^2^ = 32%, Fig. 1).

**Table 3.** AKAP9-M463I (rs6964587) and risk of breast cancer based on published and new BCAC data

Group/genotype	Controls, *N* (%)	Cases, *N* (%)	OR^^a^^ (95% CI)	*P*-value
European women				
Published data (21 studies)				
GG	12 650 (38)	8952 (37)	1.00	
GT	15 785 (47)	11 400 (47)	1.01 (0.97–1.05)	0.58
TT	4941 (15)	3802 (16)	1.09 (1.03–1.15)	0.0022
Per T-allele			1.04 (1.01–1.06)	0.0058
New COGS data (40 studies^^b^^)				
GG	11 044 (38)	11 206 (36)	1.00	
GT	13 858 (47)	14 956 (48)	1.06 (1.02–1.10)	0.0031
TT	4390 (15)	5129 (16)	1.13 (1.07–1.19)	1.6 × 10^−6^
Per T-allele			1.06 (1.04–1.09)	9.2 × 10^−7^
Asian women				
Published data (two studies)				
GG	1514 (69)	1746 (67)	1.00	
GT	615 (28)	763 (29)	1.06 (0.93–1.20)	0.58
TT	63 (2.9)	86 (3.3)	1.16 (0.83–1.62)	0.42
Per T-allele			1.07 (0.96–1.19)	0.37
New COGS data (nine studies^^c^^)				
GG	4209 (65)	3716 (65)	1.00	
GT	2012 (31)	1764 (31)	1.02 (0.94–1.11)	0.58
TT	241 (3.7)	199 (3.5)	1.03 (0.84–1.26)	0.79
Per T-allele			1.02 (0.95–1.09)	0.57
African-American women				
New COGS data (two studies)				
GG	213 (23)	299 (27)	1.00	
GT	480 (52)	531 (48)	0.80 (0.64–0.99)	0.04
TT	236 (25)	285 (26)	0.89 (0.70–1.15)	0.38
Per T-allele			0.95 (0.84–1.07)	0.39

BCAC, Breast Cancer Association Consortium; OR, odds ratio; CI, confidence interval.

^a^OR estimated by logistic regression, adjusted for study (published data); adjusted for study and principal components (new data).

^b^Nineteen studies of European women contributed both published data and new data.

^c^Two studies of Asian women contributed both published data and new data.

We also had access to the original combined data from nine GWASs used to select the majority of the BCAC SNPs on iCOGS. These included either measured or imputed genotypes for rs6964587 (4). Data for 7938 cases and 11 809 controls had not been included in the analyses conducted to date. The estimated OR based on a meta-analysis of these GWAS data was 1.05 per T-allele (95% CI = 1.01–1.10, *P* = 0.027). This model was a better fit than a recessive model (OR = 1.07, 95% CI = 1.00–1.14, *P* = 0.043). When these GWAS data were combined with the iCOGS and previously published data, the estimated per-allele OR for rs6964587 was 1.05 (95% CI = 1.04–1.07, *P* = 2.0 × 10^−10^).

The T allele of rs6964587 was less frequent in Asians (0.19) and more frequent in African-American women (0.51) than in Europeans (0.39). While there was no statistically significant evidence of association in either Asian or African-American women, the estimated OR in Asians (after combining available data, OR = 1.05, 95% CI = 0.99–1.11) was similar to that in Europeans, and in both non-European populations the 95% CIs included the OR estimate in Europeans (Table 3). Based on data for European women, there was evidence of association for both ER-positive (OR = 1.06, 95% CI = 1.04–1.08, *P* = 3.2 × 10^−8^) and ER-negative breast cancer (OR = 1.04, 95% CI = 1.01–1.07, *P* = 0.019; *P* = 0.47 for difference in OR by ER disease).There was no evidence of differences in the OR by age (*P* = 0.58), family history (*P* = 0.74) or any of the other tumor characteristics considered (PR status, HER2 status, axillary node status, grade, size or morphology; *P* ≥ 0.084).

There were no other SNPs genotyped on iCOGS within 500 kb of rs6964587 that gave stronger evidence of association in Europeans, based on the BCAC data. However, there were 133 SNPs that gave stronger evidence based on imputed genotypes (all with imputation *r*^2^ > 0.90); an intronic single-base deletion in *AKAP9* (chr7:91681597), located 51 kb from rs6964587, was the best imputed hit (*P* = 4.4 × 10^−7^, compared with 1.7 × 10^−6^ for rs6964587 in the same dataset). This variant was also well imputed in Asians and African Americans (imputation *r*^2^ = 0.99), but no independent evidence of association was observed in either (*P* > 0.35). There were three genotyped and 63 imputed (with imputation *r*^2^ > 0.8) SNPs with *P* below an arbitrary cut-off of 0.001 in Asian women, but the evidence of association for these SNPs in European women was weak (*P* ≥ 0.0029) relative to that for rs6964587.

**Figure 2.**
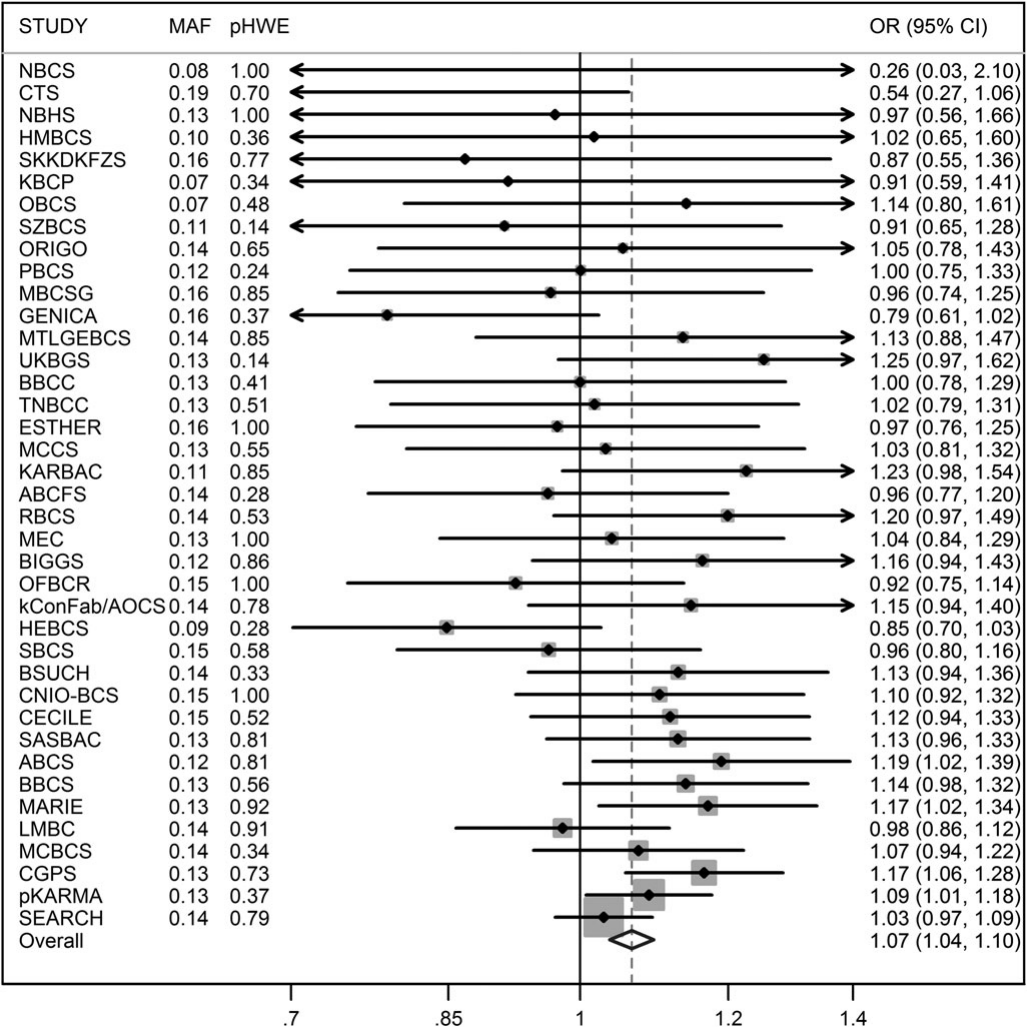
Per-allele OR estimates for *ATXN7*-K264R (rs1053338) for European women by study, based on data from the Breast Cancer Association Consortium. MAF, minor allele frequency; pHWE, *P*-value for departure from Hardy–Weinberg equilibrium; CI, confidence interval.

Results for nsSNP rs1053338 in *ATXN7* are presented in Table 4. The per-allele OR estimate for Europeans was 1.07 (95% CI = 1.04–1.10, *P* = 2.9 × 10^−6^) before, and 1.06 (95% CI = 1.03–1.09 *P* = 1.7 × 10^−5^) after, excluding 3290 cases with carcinoma *in situ* or unknown invasiveness. No notable between-study heterogeneity was observed (*I*^2^ = 14%, Fig. 2). The estimated OR based on a meta-analysis of data for the independent 8800 cases and 11 809 controls from the nine GWASs was 1.07 per T-allele (95% CI = 1.01–1.14, *P* = 0.034). A combined analysis of BCAC and GWAS data gave an estimate of 1.07 (95% CI = 1.05–1.10, *P* = 1.0 × 10^−8^).

**Table 4.** ATXN7-K264R (rs1053338) and risk of breast cancer based on BCAC data

Group/genotype	Controls, *N* (%)	Cases, *N* (%)	OR^^a^^ (95% CI)	*P*
European women				
GG	32 062 (75)	34 467 (74)	1.00	
GT	9764 (23)	11 056 (24)	1.07 (1.03–1.10)	5.6 × 10^−5^
TT	773 (1.8)	925 (2.0)	1.14 (1.04–1.26)	0.0073
Per T-allele			1.07 (1.04–1.10)	2.9 × 10^−6^
Asian women				
GG	4978 (75)	4600 (73)	1.00	
GT	1534 (23)	1536 (25)	1.03 (0.94–1.12)	0.55
TT	112 (1.7)	132 (2.1)	1.07 (0.82–1.39)	0.63
Per T-allele			1.03 (0.96–1.11)	0.46
African-American women				
GG	873 (94)	1045 (94)	1.00	
GT	59 (6.3)	70 (6.3)	0.95 (0.66–1.37)	0.80
TT	0 (0)	1 (0.0)	–	–
Per T-allele			0.97 (0.68–1.40)	0.89

COGS, Collaborative Oncological Gene-Environment Study; OR, odds ratio; CI, confidence interval.

^a^OR estimated by logistic regression, adjusted for study and principal components.

The minor T allele of rs1053338 has a similar frequency (0.13) in European and Asian women, but was much less frequent in African Americans (0.032). The results for Asian and African-American women were consistent with those for Europeans (*P-*het = 0.77; Table 4). There was no evidence of a differential association with the risk of disease subtypes defined by ER status in Europeans (*P* = 0.62); the estimated per-allele OR was 1.07 (95% CI = 1.04–1.11, *P* = 1.2 × 10^−5^) and 1.05 (95% CI = 1.00–1.11, *P* = 0.073) for ER-positive and ER-negative disease, respectively. Similar results by ER status were observed in Asian women. No evidence of heterogeneity in the OR by age was found (*P* = 0.11). We observed some evidence of a trend (*P* = 0.0075) in the associated effect size by grade, with the association only being apparent for Grade 2 and Grade 3 disease [OR = 0.98 (95% CI = 0.93–1.04) for Grade 1 disease, 1.08 (95% CI = 1.04–1.13) for Grade 2 and 1.08 (95% CI = 1.03–1.14) for Grade 3 disease]. The trend of increasing relative risk of higher grade disease was also observed for Asian women (*P* = 0.0017). There was no evidence of heterogeneity in the OR by family history (*P* = 0.66), or for any of the other tumor characteristics considered (PR status, HER2 status, axillary node status, size or morphology; *P* ≥ 0.074).

We assessed associations with other SNPs within 500 kb either side of rs1053338, both genotyped and imputed, based on BCAC iCOGS data. Slightly stronger evidence of association was observed in Europeans for one other genotyped SNP: rs3821902, an intronic variant in *ATXN7* located 26 kb away (OR = 1.08, 95% CI = 1.05–1.11, *P* = 7.4 × 10^−8^). For Asians and African Americans the *P*-value for this SNP was 0.48 and 0.54, respectively. Two other imputed SNPs (rs2241822 and rs6445387, imputation *r*^2^ ≥ 0.98), both within 5 kb of rs1053338 and both intronic to *ATXN7*, had a slightly lower *P*-value (*P* = 5.1 × 10^−8^). All three SNPs were strongly correlated with rs1053338 (*r*^2^ ≥ 0.83). No independent evidence was observed for these SNPs in the other ethnic groups (*P* > 0.31). There was only one imputed SNP with *P* < 0.01 in Asian women (rs9837159; *P* = 0.0093); the evidence of association for this SNP in European women was weak (*P* = 0.078).

## DISCUSSION

In this study of 41 non-synonymous coding SNPs, selected based on prior evidence of association with breast cancer, we have identified a novel susceptibility locus at 3p21 based on SNP rs1053338 (K264R) in *ATXN7*. We have also confirmed for the first time at genome-wide statistical significance, that *AKAP9-*rs6964587 (M463I) at 7q21 is a marker of breast cancer susceptibility in European women. In both cases, a nominally statistically significant result was observed in a meta-analysis of independent data from nine GWASs, with very similar OR estimates to those found in the BCAC COGS dataset. Both nsSNPs are associated with relatively small per-allele effects (estimated OR = 1.07 and 1.05, respectively) and appeared to confer susceptibility to ER-positive and ER-negative disease. The potentially differential association of rs1053338 with risk of breast cancer by grade requires confirmation.

That independent confirmation of these associations was not observed for Asian and African-American women may be explained by the limited power to detect these effect sizes. We estimate that at 5% statistical significance our study had <50% power to detect the ORs estimated for European women for these SNPs in Asian women and much lower power (<15%) for African-American women. However, weaker associations in non-European populations have been observed for many breast cancer susceptibility loci and may reflect differences in LD patterns, genetic background and/or the distribution of interacting environmental risk factors.

The nsSNP giving the strongest signal in our study was rs10510592 (L513S) in *NEK10*, located within an established breast cancer susceptibility region. However, substantially stronger evidence of association with risk was observed for the originally reported SNP at this locus (rs4973768), and further analyses revealed that the association with rs10510592 was substantially attenuated after adjusting for rs4973768. Hence, if there is a single causal variant in this region, it is unlikely to be rs10510592, despite the fact that this SNP is an amino acid substitution with strong evidence of association with disease risk (*P* = 5.1 × 10^−17^). Further work, including *in vitro* analyses to functionally characterize candidate variants, will be required identify to the biological mechanism behind this clear association.

The same phenomenon was observed for the two nsSNPs marking novel breast cancer susceptibility loci that we have identified in the present study. In both cases, the nsSNP could not be definitively ruled out as the causal variant. Nevertheless, in the case of *ATXN7-*K264R, three intronic SNPs in the same gene, one genotyped and two imputed, gave stronger signals of association. Similarly, while *AKAP9-*M463I gave the strongest signal among the genotyped SNPs, an imputed intronic SNP had an associated *P*-value almost an order of magnitude smaller. Future studies that fine-map these two regions through dense genotyping, in even larger sample sizes, will therefore be required to identify the casual variants and targeted genes.

The WTCCC also noted that an observed association with an nsSNP does not necessarily imply that the SNP, or even the gene in which it is located, is causal (22). That is, a candidate variant approach may identify novel susceptibility loci, but the variant in question cannot be assumed to be causal, highlighting the importance of rigorous fine-scale mapping analyses, even when an association with a potentially functional SNP has been identified. These results are also consistent with previous observations that the vast majority of common susceptibility alleles for breast cancer are non-coding; even after deliberately selecting potentially associated nsSNPs, the confirmed associations appear to be markers for other, presumably non-coding, functional SNPs.

For both the *AKAP9* and *ATXN7* nsSNPs, a consistent association was observed in the BCAC dataset and the combined analysis of nine GWASs. It is interesting to note, however, that neither locus was selected for inclusion on the iCOGS array based on evidence of association in the combined GWAS, despite the fact that the array included >35 273 SNPs selected for replication of the GWAS (4); both loci failed to reach the cut-off of *P* < 0.008. Indeed, the probability that loci with associated effects of this magnitude would have been selected for inclusion on iCOGS on the basis of their GWAS-based results was <0.40. These results emphasize that, for associations of this magnitude (OR = 1.05–1.07), even a combined GWAS of >10 000 cases and 10 000 controls has limited power. They also highlight that further loci with associated effects of similar magnitude remain to be identified (4).

A key strength of this study is the sample size; the iCOGS study is the largest genotyping study in breast cancer, and by far the largest study to evaluate non-synonymous SNPs. There is potentially some overlap between the samples used in the WTCCC study and the current analysis. The WTCCC study used samples from a UK study of familial breast cancer (FBCS) that was also used in one of the GWAS (UK2). Although it is not possible to check directly, any overlap with the samples used in the COGS would have been incidental: we estimate that <3% of samples in the BCAC COGS analysis could have been used in the WTCCC analysis. Moreover, since both loci reach genome-wide levels of significance, the evidence for these associations being real does not depend strongly on their selection through the WTCCC study.

In summary, in this very large case–control study focused on common candidate non-synonymous variants, we have identified a novel susceptibility locus at 3p21 and confirmed *AKAP9-*rs6964587 as a marker of a breast cancer risk at 7q21. Additional analyses of other common variants in these regions, the majority imputed from the 1000 genomes project, suggest that the nsSNPs genotyped are unlikely to be causal and that further fine-mapping studies are required to identify the variants and corresponding genes that modify breast cancer risk.

## MATERIALS AND METHODS

### Participants

Samples for the main study were drawn from 49 case–control studies participating in the BCAC (Table 1): 38 from populations of predominantly European ancestry (46 450 cases and 42 600 controls), nine from populations of Asian ancestry (6269 cases and 6624 controls) and two of African-American women (1116 cases and 932 controls). Studies were either population based or hospital based; some studies sampled cases according to age, or oversampled for cases with a family history or bilateral disease (Supplementary Material, Table S1). All study participants gave informed consent and all studies were approved by the corresponding local ethics committees.

### SNP selection

We considered the 48 SNPs for which the strongest evidence of association (per-allele test *P*-value < 0.005) with breast cancer was observed in the original analysis by the WTCCC (22). In addition, we considered an nsSNP in *AKAP9* based on previous evidence from the BCAC (23,24) and for which consistent results were reported in the WTCCC study, even though the *P*-value did not meet the 0.005 threshold (22). Pairwise LD was assessed based on the correlation coefficient (*r*^2^) in Europeans from HapMap data release 28 (Phases II and III) and visualised using Haploview version 4.2. Two nsSNPs (rs4148077 and rs4986791) were in complete LD (*r*^2^ = 1.0) with other variants considered (rs3742801 and rs4986790, respectively) and were therefore excluded. A further three SNPs (rs11465716, rs3790549 and rs7313899) were excluded because they were reported to have an MAF < 5%. Genotyping assays could not be designed for nine SNPs (Illumina design score <0.8), but surrogate SNPs could be genotyped for six of these, five in complete LD with the original SNP and one in high LD (*r*^2^ = 0.94); the remaining three SNPs (rs4255378, rs2074491 and rs4730283) could not be assessed. The 41 SNPs considered in this analysis are listed in Table 2 and their selection is summarized in the Supplementary Material, Fig. S1.

### Genotyping

Genotyping was conducted using a custom Illumina Infinium array (iCOGS) in four centers, as part of the COGS, as described previously (4). Genotypes were called using Illumina's proprietary GenCall algorithm. QC procedures have been previously described (4). Subjects with an overall call-rate <95% were excluded. Genotype intensity cluster plots were checked manually for SNPs for which evidence of association at *P* < 0.0001 was found, and all were judged to be acceptable, with the exception of that for rs6964587. However, clearly defined clusters were observed for rs6964587 after excluding 1259 samples from plates with call-rates <90% and all subsequent analyses for this SNP were based on this slightly reduced sample.

### Statistical methods

Ethnic outliers were identified by multi-dimensional scaling, combining the iCOGS data with the three Hapmap2 populations, based on a subset of 37 000 uncorrelated markers that passed QC (including ∼1000 selected as ancestry informative markers). Most studies were predominantly of a single ancestry (European or Asian), and individuals with >15% minority ancestry, based on the first two components, were excluded. Exceptions to this were the two studies of African Americans (NBHS and SCCS) and two of the Asian studies, from Singapore (SGBCC) and Malaysia (MYBRCA), which contained a substantial fraction of individuals of mixed ancestry and so no exclusions were made based on genetically determined ethnicity. Principal components analyses were then carried out separately for the European, Asian and African-American subgroups, based on the same subset of SNPs. Results presented are for women of European ancestry, unless otherwise stated.

Departure from Hardy–Weinberg equilibrium (HWE) was tested for in controls using a study-stratified *χ*^2^ test (1 d.f.) (25,26). The association of each SNP with breast cancer risk was assessed by estimating genotype-specific and per-allele ORs using logistic regression, adjusted for study. For the analyses of European women, we also included the first six principal components as covariates, together with a seventh component specific to one study (LMBC) for which there was substantial inflation not accounted for by the components derived from the analysis of all studies. The inclusion of additional principal components did not reduce inflation further. We included two race-specific principal components in the analyses of Asian and African-American women.

Between-study heterogeneity in ORs was assessed for each of the three broad racial groups using the *metan* command in Stata (Release 10) (27) to meta-analyse study-specific per-allele log-OR estimates and generate *I*^2^ statistics; values >50% were considered notable (28). Differences in ORs by ethnicity were assessed using a likelihood ratio test (LRT) comparing the model with interaction terms for the per-allele log-OR by study population (European, Asian, African American) to the model with no interaction terms. Differences by age (<40, 40–49, 50–59, 60–69 and ≥70 years) were evaluated using a similar LRT, but modeling a linear trend by fitting the median age for each of these defined categories.

Heterogeneity in the OR by first degree family history (no, yes), by subtypes defined by ER, PR and HER2 status (positive, negative) and by axillary node status (none, ≥1 affected), tumor grade (1–3), tumor size (≤10, 11–20, >20 mm) and tumor morphology (ductal, lobular), was assessed by applying polytomous logistic regression to cases only, with the number of rare alleles as the outcome and restricting, for each explanatory variable, the beta coefficient for the comparison of 2–0 minor alleles to be double that for the comparison of 1–0 minor alleles. Linear trends were tested by fitting as continuous variables values 1, 2 and 3 for grade and the median value for each the defined categories of size. ORs specific to disease subtypes defined by ER status were estimated for Europeans using polytomous logistic regression with control status as the reference outcome. All statistical tests were two sided. The term ‘genome-wide statistically significant’ is taken to imply *P* < 5 × 10^−8^; otherwise ‘statistically significant’ implies *P* < 0.05. Power calculations were carried out using Quanto v.1.2.4 (http://biostats.usc.edu/software). All other analyses were conducted using Stata: release 10 (27). The analysis pipeline is summarized in the Supplementary Material, Fig. S1.

Genotype data for iCOGS SNPs in regions surrounding rs6864587 and rs1053338 were used to estimate genotypes for other common variants across those regions for the BCAC study subjects by imputation, using IMPUTE v2.2 and the March 2012 release of the 1000 Genomes Project as reference panel. SNPs with an imputation *r*^2^ < 0.80 were excluded.

## SUPPLEMENTARY MATERIAL

Supplementary Material is available at *HMG* online.

## FUNDING

BCAC is funded by Cancer Research UK (C1287/A10118, C1287/A12014) and by the European Community's Seventh Framework Programme under grant agreement n° 223175 (HEALTH-F2–2009-223175) (COGS). Meetings of the BCAC have been funded by the European Union COST programme (BM0606). Genotyping of the iCOGS array was funded by the European Union (HEALTH-F2-2009-223175), Cancer Research UK (C1287/A10710), the Canadian Institutes of Health Research for the ‘CIHR Team in Familial Risks of Breast Cancer’ program and the Ministry of Economic Development, Innovation and Export Trade of Quebec (PSR-SIIRI-701). Additional support for the iCOGS infrastructure was provided by the National Institutes of Health (CA128978) and Post-Cancer GWAS initiative (1U19 CA148537, 1U19 CA148065 and 1U19 CA148112—the GAME-ON initiative), the Department of Defence (W81XWH-10-1-0341), Komen Foundation for the Cure, the Breast Cancer Research Foundation, and the Ovarian Cancer Research Fund. The ABCFS and OFBCR work was supported by grant UM1 CA164920 from the National Cancer Institute (USA). The content of this manuscript does not necessarily reflect the views or policies of the National Cancer Institute or any of the collaborating centers in the Breast Cancer Family Registry (BCFR), nor does mention of trade names, commercial products or organizations imply endorsement by the US Government or the BCFR. The ABCFS was also supported by the National Health and Medical Research Council of Australia, the New South Wales Cancer Council, the Victorian Health Promotion Foundation (Australia) and the Victorian Breast Cancer Research Consortium. J.L.H. is a National Health and Medical Research Council (NHMRC) Senior Principal Research Fellow and M.C.S. is a NHMRC Senior Research Fellow. The OFBCR work was also supported by the Canadian Institutes of Health Research ‘CIHR Team in Familial Risks of Breast Cancer’ program. The ABCS was funded by the Dutch Cancer Society Grant no. NKI2007-3839 and NKI2009-4363. The ACP study is funded by the Breast Cancer Research Trust, UK. The work of the BBCC was partly funded by ELAN-Programme of the University Hospital of Erlangen. The BBCS is funded by Cancer Research UK and Breakthrough Breast Cancer and acknowledges NHS funding to the NIHR Biomedical Research Centre, and the National Cancer Research Network (NCRN). E.S. is supported by NIHR Comprehensive Biomedical Research Centre, Guy's & St. Thomas’ NHS Foundation Trust in partnership with King's College London, UK. Core funding to the Wellcome Trust Centre for Human Genetics was provided by the Wellcome Trust (090532/Z/09/Z). I.T. is supported by the Oxford Biomedical Research Centre. The BSUCH study was supported by the Dietmar-Hopp Foundation, the Helmholtz Society and the German Cancer Research Center (DKFZ). The CECILE study was funded by the Fondation de France, the French National Institute of Cancer (INCa), The National League against Cancer, the National Agency for Environmental and Occupational Health and Food Safety (ANSES), the National Agency for Research (ANR), and the Association for Research against Cancer (ARC). The CGPS was supported by the Chief Physician Johan Boserup and Lise Boserup Fund, the Danish Medical Research Council and Herlev Hospital. The CNIO-BCS was supported by the Genome Spain Foundation, the Red Temática de Investigación Cooperativa en Cáncer and grants from the Asociación Española Contra el Cáncer and the Fondo de Investigación Sanitario (PI11/00923 and PI081120). The Human Genotyping-CEGEN Unit, CNIO is supported by the Instituto de Salud Carlos III. D.A. was supported by a Fellowship from the Michael Manzella Foundation (MMF) and was a participant in the CNIO Summer Training Program. The CTS was initially supported by the California Breast Cancer Act of 1993 and the California Breast Cancer Research Fund (contract 97-10500) and is currently funded through the National Institutes of Health (R01 CA77398). Collection of cancer incidence data was supported by the California Department of Public Health as part of the statewide cancer reporting program mandated by California Health and Safety Code Section 103885. HAC receives support from the Lon V Smith Foundation (LVS39420). The ESTHER study was supported by a grant from the Baden Württemberg Ministry of Science, Research and Arts. Additional cases were recruited in the context of the VERDI study, which was supported by a grant from the German Cancer Aid (Deutsche Krebshilfe). The GENICA was funded by the Federal Ministry of Education and Research (BMBF) Germany grants 01KW9975/5, 01KW9976/8, 01KW9977/0 and 01KW0114, the Robert Bosch Foundation, Stuttgart, Deutsches Krebsforschungszentrum (DKFZ), Heidelberg, Institute for Prevention and Occupational Medicine of the German Social Accident Insurance, Institute of the Ruhr University Bochum (IPA), as well as the Department of Internal Medicine, Evangelische Kliniken Bonn gGmbH, Johanniter Krankenhaus Bonn, Germany. The HEBCS was supported by the Helsinki University Central Hospital Research Fund, Academy of Finland (132473), the Finnish Cancer Society, The Nordic Cancer Union and the Sigrid Juselius Foundation. The HERPACC was supported by a Grant-in-Aid for Scientific Research on Priority Areas from the Ministry of Education, Science, Sports, Culture and Technology of Japan, by a Grant-in-Aid for the Third Term Comprehensive 10-Year Strategy for Cancer Control from Ministry Health, Labour and Welfare of Japan, by a research grant from Takeda Science Foundation, by Health and Labour Sciences Research Grants for Research on Applying Health Technology from Ministry Health, Labour and Welfare of Japan and by National Cancer Center Research and Development Fund. The HMBCS was supported by short-term fellowships from the German Academic Exchange Program (to N.B), and the Friends of Hannover Medical School (to N.B.). Financial support for KARBAC was provided through the regional agreement on medical training and clinical research (ALF) between Stockholm County Council and Karolinska Institutet, the Stockholm Cancer Foundation and the Swedish Cancer Society. The KBCP was financially supported by the special Government Funding (EVO) of Kuopio University Hospital grants, Cancer Fund of North Savo, the Finnish Cancer Organizations, the Academy of Finland and by the strategic funding of the University of Eastern Finland. kConFab is supported by grants from the National Breast Cancer Foundation, the NHMRC, the Queensland Cancer Fund, the Cancer Councils of New South Wales, Victoria, Tasmania and South Australia and the Cancer Foundation of Western Australia. The kConFab Clinical Follow Up Study was funded by the NHMRC (145684, 288704, 454508). Financial support for the AOCS was provided by the United States Army Medical Research and Materiel Command (DAMD17-01-1-0729), the Cancer Council of Tasmania and Cancer Foundation of Western Australia and the NHMRC (199600). G.C.T. and P.W. are supported by the NHMRC. LAABC is supported by grants (1RB-0287, 3PB-0102, 5PB-0018 and 10PB-0098) from the California Breast Cancer Research Program. Incident breast cancer cases were collected by the USC Cancer Surveillance Program (CSP) which is supported under subcontract by the California Department of Health. The CSP is also part of the National Cancer Institute's Division of Cancer Prevention and Control Surveillance, Epidemiology, and End Results Program, under contract number N01CN25403. LMBC is supported by the ‘Stichting tegen Kanker’ (232-2008 and 196-2010). The MARIE study was supported by the Deutsche Krebshilfe e.V. (70-2892-BR I), the Federal Ministry of Education and Research (BMBF) Germany (01KH0402), the Hamburg Cancer Society and the German Cancer Research Center (DKFZ). MBCSG is supported by grants from the Italian Association for Cancer Research (AIRC) and by funds from the Italian citizens who allocated a 5/1000 share of their tax payment in support of the Fondazione IRCCS Istituto Nazionale Tumori, according to Italian laws (INT-Institutional strategic projects ‘5 × 1000’). The MCBCS was supported by the NIH grants (CA122340, CA128978) and a Specialized Program of Research Excellence (SPORE) in Breast Cancer (CA116201), the Breast Cancer Research Foundation and a generous gift from the David F. and Margaret T. Grohne Family Foundation and the Ting Tsung and Wei Fong Chao Foundation. MCCS cohort recruitment was funded by VicHealth and Cancer Council Victoria. The MCCS was further supported by Australian NHMRC grants 209057, 251553 and 504711 and by infrastructure provided by Cancer Council Victoria. The MEC was supported by NIH grants CA63464, CA54281, CA098758 and CA132839. The work of MTLGEBCS was supported by the Quebec Breast Cancer Foundation, the Canadian Institutes of Health Research (grant CRN-87521) and the Ministry of Economic Development, Innovation and Export Trade (grant PSR-SIIRI-701). MYBRCA is funded by research grants from the Malaysian Ministry of Science, Technology and Innovation (MOSTI), Malaysian Ministry of Higher Education (UM.C/HlR/MOHE/06) and Cancer Research Initiatives Foundation (CARIF). Additional controls were recruited by the Singapore Eye Research Institute, which was supported by a grant from the Biomedical Research Council (BMRC08/1/35/19<tel:08/1/35/19>/550), Singapore and the National medical Research Council, Singapore (NMRC/CG/SERI/2010). The NBCS was supported by grants from the Norwegian Research council (155218/V40, 175240/S10 to A.L.B.D., FUGE-NFR 181600/V11 to V.N.K. and a Swizz Bridge Award to A.L.B.D.). The NBHS was supported by NIH grant R01CA100374. Biological sample preparation was conducted the Survey and Biospecimen Shared Resource, which is supported by P30 CA68485. The OBCS was supported by research grants from the Finnish Cancer Foundation, the Sigrid Juselius Foundation, the Academy of Finland, the University of Oulu, and the Oulu University Hospital. The ORIGO study was supported by the Dutch Cancer Society (RUL 1997-1505) and the Biobanking and Biomolecular Resources Research Infrastructure (BBMRI-NL CP16). The PBCS was funded by Intramural Research Funds of the National Cancer Institute, Department of Health and Human Services, USA. pKARMA is a combination of the KARMA and LIBRO-1 studies. KARMA was supported by Märit and Hans Rausings Initiative Against Breast Cancer. KARMA and LIBRO-1 were supported the Cancer Risk Prediction Center (CRisP; www.crispcenter.org), a Linnaeus Centre (Contract ID 70867902) financed by the Swedish Research Council. The RBCS was funded by the Dutch Cancer Society (DDHK 2004-3124, DDHK 2009-4318). SASBAC was supported by funding from the Agency for Science, Technology and Research of Singapore (A*STAR), the US National Institute of Health (NIH) and the Susan G. Komen Breast Cancer Foundation. KC was financed by the Swedish Cancer Society (5128-B07-01PAF). The SBCGS was supported primarily by NIH grants R01CA64277, R01CA148667, and R37CA70867. Biological sample preparation was conducted the Survey and Biospecimen Shared Resource, which is supported by P30 CA68485. The SBCS was supported by Yorkshire Cancer Research S305PA, S299 and S295. Funding for the SCCS was provided by NIH grant R01 CA092447. The Arkansas Central Cancer Registry is fully funded by a grant from National Program of Cancer Registries, Centers for Disease Control and Prevention (CDC). Data on SCCS cancer cases from Mississippi were collected by the Mississippi Cancer Registry which participates in the National Program of Cancer Registries (NPCR) of the Centers for Disease Control and Prevention (CDC). The contents of this publication are solely the responsibility of the authors and do not necessarily represent the official views of the CDC or the Mississippi Cancer Registry. SEARCH is funded by a programme grant from Cancer Research UK (C490/A10124) and supported by the UK National Institute for Health Research Biomedical Research Centre at the University of Cambridge. The SEBCS was supported by the BRL (Basic Research Laboratory) program through the National Research Foundation of Korea funded by the Ministry of Education, Science and Technology (2012-0000347). SGBCC is funded by the National Medical Research Council Start-up Grant and Centre Grant (NMRC/CG/NCIS /2010). The recruitment of controls by the Singapore Consortium of Cohort Studies-Multi-ethnic cohort (SCCS-MEC) was funded by the Biomedical Research Council (grant number: 05/1/21/19/425). SKKDKFZS is supported by the DKFZ. The SZBCS was supported by Grant PBZ_KBN_122/P05/2004. K. J. is a fellow of International PhD program, Postgraduate School of Molecular Medicine, Warsaw Medical University, supported by the Polish Foundation of Science. The TNBCC was supported by the NIH grant (CA128978), the Breast Cancer Research Foundation, Komen Foundation for the Cure, the Ohio State University Comprehensive Cancer Center, the Stefanie Spielman Fund for Breast Cancer Research and a generous gift from the David F. and Margaret T. Grohne Family Foundation and the Ting Tsung and Wei Fong Chao Foundation. Part of the TNBCC (DEMOKRITOS) has been co-financed by the European Union (European Social Fund – ESF) and Greek National Funds through the Operational Program ‘Education and Lifelong Learning’ of the National Strategic Reference Framework (NSRF)—Research Funding Program of the General Secretariat for Research & Technology: ARISTEIA. The TWBCS is supported by the Institute of Biomedical Sciences, Academia Sinica and the National Science Council, Taiwan. The UKBGS is funded by Breakthrough Breast Cancer and the Institute of Cancer Research (ICR). ICR acknowledges NHS funding to the NIHR Biomedical Research Centre. Funding to pay the Open Access publication charges for this article was provided by the Wellcome Trust.

## Supplementary Material

Supplementary Data
