# Supplementary material for: Common non-synonymous SNPs associated with breast cancer susceptibility: findings from the Breast Cancer Association Consortium
Source: Hum Mol Genet. 2014 Jun 18;23(22):6096–111. doi: 10.1093/hmg/ddu311 (PMC4204770; doi:10.1093/hmg/ddu311)
Supplement: Supplementary Data [file supp_23_22_6096__index.html]

Common non-synonymous SNPs associated with breast cancer susceptibility: findings from the Breast Cancer Association Consortium — Common non-synonymous SNPs associated with breast cancer susceptibility: findings from the Breast Cancer Association Consortium — Supplementary Data 

# Common non-synonymous SNPs associated with breast cancer susceptibility: findings from the Breast Cancer Association Consortium

## Supplementary Data

Supplementary Data

**Files in this Data Supplement:**

- Supplementary Figure 1 - pptx file
- Supplementary Table 1 - docx file
