# Supplementary material for: Common non-synonymous SNPs associated with breast cancer susceptibility: findings from the Breast Cancer Association Consortium
Source: Hum Mol Genet. 2014 Jun 18;23(22):6096–111. doi: 10.1093/hmg/ddu311 (PMC4204770; doi:10.1093/hmg/ddu311)
Supplement: Supplementary Data [file supp_ddu311_ddu311supp_fig1.pptx]

## Slide 1
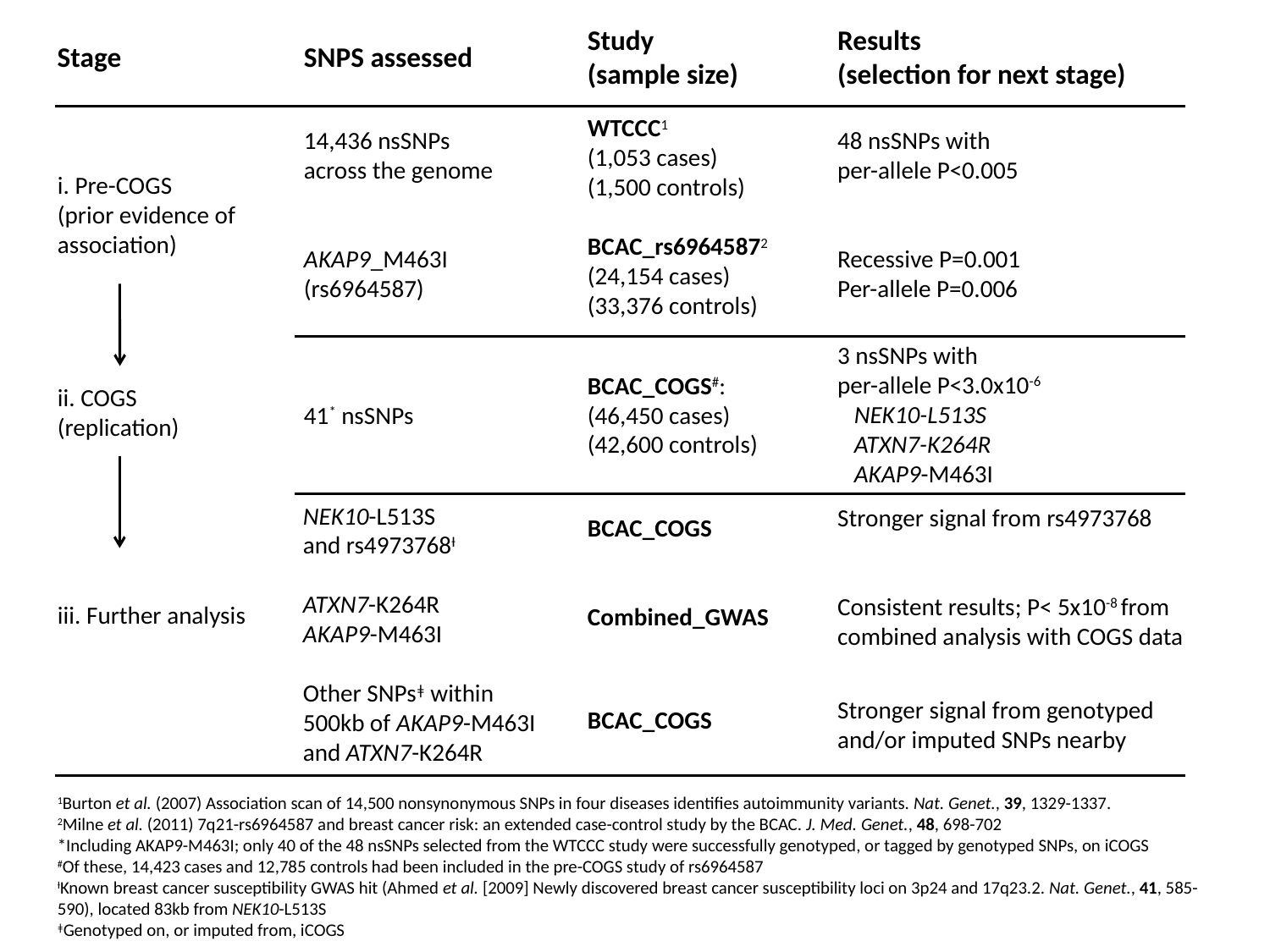

Stage
SNPS assessed
Study
(sample size)
Results
(selection for next stage)
WTCCC1
(1,053 cases)
(1,500 controls)
BCAC_rs69645872
(24,154 cases)
(33,376 controls)
14,436 nsSNPs across the genome
AKAP9_M463I
(rs6964587)
48 nsSNPs with
per-allele P<0.005
Recessive P=0.001
Per-allele P=0.006
i. Pre-COGS
(prior evidence of association)
3 nsSNPs with
per-allele P<3.0x10^-6^
 NEK10-L513S
 ATXN7-K264R
 AKAP9-M463I
BCAC_COGS#:
(46,450 cases)
(42,600 controls)
ii. COGS
(replication)
41* nsSNPs
 NEK10-L513S
 and rs4973768Ɨ
 ATXN7-K264R
 AKAP9-M463I
 Other SNPsǂ within
 500kb of AKAP9-M463I
 and ATXN7-K264R
Stronger signal from rs4973768
Consistent results; P< 5x10^-8^ from combined analysis with COGS data
Stronger signal from genotyped and/or imputed SNPs nearby
BCAC_COGS
Combined_GWAS
BCAC_COGS
iii. Further analysis
1Burton et al. (2007) Association scan of 14,500 nonsynonymous SNPs in four diseases identifies autoimmunity variants. Nat. Genet., 39, 1329-1337.
2Milne et al. (2011) 7q21-rs6964587 and breast cancer risk: an extended case-control study by the BCAC. J. Med. Genet., 48, 698-702
*Including AKAP9-M463I; only 40 of the 48 nsSNPs selected from the WTCCC study were successfully genotyped, or tagged by genotyped SNPs, on iCOGS
#Of these, 14,423 cases and 12,785 controls had been included in the pre-COGS study of rs6964587
ƗKnown breast cancer susceptibility GWAS hit (Ahmed et al. [2009] Newly discovered breast cancer susceptibility loci on 3p24 and 17q23.2. Nat. Genet., 41, 585-590), located 83kb from NEK10-L513S
ǂGenotyped on, or imputed from, iCOGS
