# Supplementary material for: Common non-synonymous SNPs associated with breast cancer susceptibility: findings from the Breast Cancer Association Consortium
Source: Hum Mol Genet. 2014 Jun 18;23(22):6096–111. doi: 10.1093/hmg/ddu311 (PMC4204770; doi:10.1093/hmg/ddu311)
Supplement: Supplementary Data [file supp_ddu311_ddu311supp_table1.docx]

**Supplementary Table 1.** Description of the BCAC studies contributing to COGS.

| **Study Acronym** | **Study Name**  **(Reference)** | **Country** | **Recruitment base** | |
| --- | --- | --- | --- | --- |
|  |  |  | **Cases** | **Controls** |
| ABCFS^†^ | Australian Breast Cancer Family Study [[1](#_ENREF_1)] | Australia | Cancer registries in Victoria and New South Wales (1992-1999): all cases from Melbourne and Sydney diagnosed before age 40 plus a random sample of those diagnosed at ages 40-59. | Identified between 1992 and 1999 from the electoral rolls in Melbourne and Sydney (enrolling to vote is compulsory); frequency matched to cases by age in-5 year categories. |
| ABCS | Amsterdam Breast Cancer Study [[2](#_ENREF_2)] | Netherlands | Breast cancer patients diagnosed before age 50 in 2003-2009 at the NKI-AVL; and (ABCS-F) non-*BRCA1/2* breast cancer cases from the family cancer clinic of the NKI-AVL tested in the period 1995-2009, of all ages and diagnosed with breast cancer in 1965-2008. | Population-based cohort of women recruited through the Sanquin blood bank, all ages. |
| BBCC | Bavarian Breast Cancer Cases and Controls [[3](#_ENREF_3)] | Germany | Consecutive, unselected cases with invasive breast cancer recruited at the University Breast Centre, Franconia in Northern Bavaria from 2002-2010. | Healthy women aged 55 or older with no diagnosis of cancer. Invited by a newspaper advertisement in Northern Bavaria between 2002-2010. |
| BBCS | British Breast Cancer Study [[4](#_ENREF_4)] | U.K. | (i) English & Scottish Cancer Registries: all breast cancer cases who developed a first primary before age 65 in 1971 or later and who subsequently developed a second primary cancer;  (ii) Unilateral breast cancer cases diagnosed before age 70 in 1971 or later. | A friend, sister-in-law, daughter-in-law or other non-blood relative of cases, recruited from 2001-2008. |
| BIGGS | Breast Cancer in Galway Genetic Study [[5](#_ENREF_5)] | Ireland | Unselected cases recruited from University College Hospital Galway and surrounding hospitals in the West of Ireland since 2001 | Women > 60 years with no personal history of any cancer and no family history of breast or ovarian cancer identified from retirement groups in the West of Ireland between 2001-2008. |

| BSUCH | Breast Cancer Study of the University Clinic Heidelberg [[6](#_ENREF_6)] | Germany | All cases diagnosed with breast cancer in 2007-2009 at the University Women`s Clinic Heidelberg. | Female blood donors recruited in 2007- 2009 at the Institute of Transfusion Medicine & Immunology, Mannheim. |
| --- | --- | --- | --- | --- |
| CECILE | CECILE Breast Cancer Study [[7](#_ENREF_7)] | France | All cases diagnosed with breast cancer in 2005-2007 among women <75 years of age residing in the *départements* of Ille-et-Vilaine and Côte d'Or . Cases were recruited from the main cancer treatment center (Centre Eugène-Marquis in Rennes and Centre Georges-François-Leclerc in Dijon) and from other private or public hospitals in each area. | General population control women residing in the same areas as the cases (Ille-et-Vilaine and Côte d’Or). Controls were frequency-matched to the cases by 5-year age groups. They were recruited in 2005-2007 using a random digit dialing procedure and quotas by socioeconomic status to reflect the distribution by SES of the population in each area. |
| CGPS | Copenhagen General Population Study [[8](#_ENREF_8)] | Denmark | Consecutive, incident cases from one hospital with centralized care for a population of 400,000 women in Copenhagen (2001-present). | Women with no history of breast cancer residing in the same region as cases identified from the Copenhagen General Population Study (2003-2007). |
| CNIO-BCS | Spanish National Cancer Centre Breast Cancer Study [[9](#_ENREF_9)] | Spain | (i) consecutive breast cancer patients from three public hospitals, two in Madrid and one in Oviedo;  (ii) cases with at least one affected first degree relative recruited through the CNIO family cancer clinic in Madrid (2000-2005). | Women attending the Menopause Research Centre, Madrid and female members of the College of Lawyers attending a free, targeted medical check-up in Madrid between 2000 and 2005, all free of breast cancer. |
| CTS* | California Teachers  Study [[10](#_ENREF_10)] | U.S.A. | Nested case-control study conducted within a cohort of California teachers (113,590) who were under age 80 years at baseline, had no prior history of invasive or *in situ* breast cancer. Cases are women newly diagnosed with a histologically confirmed invasive primary adenocarcinoma of the breast at age 80 years or younger from 1998 to 2008. | Controls are a probability sample of at-risk cohort members, frequency matched to cases on age at baseline (5-year age groups), self-reported race/ethnicity (white, African American, Latina,Asian, other), and broad geographic region within California Controls were selected without replacement, using an assigned reference date. |
| ESTHER | ESTHER Breast Cancer Study [[11](#_ENREF_11)] | Germany | Breast cancer cases in all hospitals in the state of Saarland, from 2001-2003 (ESTHER) and 1996-1998 (VERDI). | Random sample of women undergoing a routine health check-up in Saarland, in 2000-2002; frequency matched to cases by age in-5 year categories. |

| GENICA* | Gene Environment Interaction & Breast Cancer in Germany [[12](#_ENREF_12),[13](#_ENREF_13)] | Germany | Incident breast cancer cases enrolled at hospitals  in the Greater Bonn area between 2000-2004. | Random address sample selected in 2001-2004 from 31 population registries in the greater Bonn area; frequency matched to cases on year of birth in 5-year categories. |
| --- | --- | --- | --- | --- |
| HEBCS | Helsinki Breast Cancer Study [[14](#_ENREF_14)] | Finland | (1) Consecutive cases (883) from the Department of Oncology, Helsinki University Central Hospital 1997-8 and 2000, (2) Consecutive cases (986) from the Department of Surgery, Helsinki University Central Hospital 2001 – 2004, (3) Familial breast cancer patients (536) from the Helsinki University Central Hospital, Departments of Oncology and Clinical Genetics (1995-). | Healthy females from the same geographical region in Southern Finland in 2003. |
| HMBCS | Hannover-Minsk Breast Cancer Study [[15](#_ENREF_15)] | Belarus | Cases from the Byelorussian Institute for Oncology and Medical Radiology Aleksandrov N.N. in Minsk or at one of 5 regional oncology centers in Gomel, Mogilev, Grodno, Brest or Vitebsk (2002-2008). | Women attending general medical examination at gynecology clinics in Gomel, Mogilev, Grodno, Brest or Vitebsk; women attending the Institute for Inherited Diseases in Minsk; female blood donors in Minsk; healthy relatives of cases (2002-2008). |
| KARBAC | Karolinska Breast Cancer Study [[16](#_ENREF_16)] | Sweden | 1. Familial cases from Department of Clinical Genetics, Karolinska University Hospital, Stockholm.  2. Consecutive cases from Department of Oncology, Huddinge & Söder Hospital, Stockholm 1998-2000. | Blood donors of mixed gender from same geographical region. Excess material was received from all blood donors over a 3 month period in 2004 (approximately 3000) and DNA was extracted from a random sample of 1500. |
| KBCP | Kuopio Breast Cancer Project [[17](#_ENREF_17)] | Finland | Women seen at Kuopio University Hospital between 1990-1995 because of a breast lump, mammographic abnormality, or other breast symptom and who were found to have breast cancer. | Selected from the National Population Register between 1990-1995; age and long-term area-of-residence matched to cases. |
| kConFab/  AOCS | Kathleen Cuningham Foundation Consortium for Research into Familial Breast Cancer / Australian Ovarian Cancer Study [[18](#_ENREF_18)] | Australia | Index (youngest affected) cases from *BRCA1*- and *BRCA2*-mutation-negative multiple-case breast and breast-ovarian families recruited though family cancer clinics from across Australia and New Zealand from 1998-present. | Identified from the electoral rolls from across Australia as part of the Australian Ovarian Cancer Study in 2002-2006. |
| LMBC | Leuven Multidisciplinary Breast Centre [[19](#_ENREF_19)] | Belgium | All patients diagnosed with breast cancer and seen in the Multidisciplinary Breast Center in Leuven (Gashuisberg) since June 2007 plus retrospective collection of cases diagnosed since 2000. | Blood donors at Gasthuisberg Hospital (200-2008). |
| MARIE | Mammary Carcinoma Risk Factor Investigation [[20](#_ENREF_20)] | Germany | Incident cases diagnosed from 2001-2005 in the study region Hamburg in Northern Germany, and from 2002-2005 in the study region Rhein-Neckar-Karlsruhe in Southern Germany. | Two controls per case were randomly drawn from population registries and frequency matched by birth year and study region to the case. Controls were recruited from 2002 to 2006. |
| MBCSG | Milan Breast Cancer Study Group [[21](#_ENREF_21)] | Italy | Familial and/or early onset breast cancer patients (aged 22-87) negative for mutations in *BRCA1* and *BRCA2*, ascertained at two large cancer centers in Milan from 2000-present. | Female blood donors recruited at two centres in Milan from 2004-present and 2007-present. |
| MCBCS | Mayo Clinic Breast Cancer Study [[22](#_ENREF_22)] | U.S.A. | Incident cases residing in 6 states (MN, WI, IA, IL, ND, SD) seen at the Mayo Clinic in Rochester, MN from 2002-2010. | Women presenting for general medical examination at the Mayo Clinic from 2002-2010; frequency matched to cases on age, ethnicity and county/state. |
| MCCS | Melbourne Collaborative Cohort Study [[23](#_ENREF_23)] | Australia | Incident cases from the cohort of 24,469 women, diagnosed during the follow-up from baseline (1990-1994) to 2008. | Random sample of the initial cohort. |
| MEC | Multiethnic Cohort [[24](#_ENREF_24)] | U.S.A. | Incident cases identified from SEER cancer registries in Los Angeles County & State registries in California & Hawaii, USA from 1993-2002. Grouped by self-reported ethnicity. | Women without cancer from the same States, recruited concurrently with cases & frequency matched to cases by age at blood-draw & self-reported ethnicity. |
| MTLGEBCS | Montreal Gene-Environment Breast Cancer Study [[25](#_ENREF_25)] | Canada | All cases are postmenopausal women (47-75 years) living in Montreal with a primary invasive breast cancer and with no previous occurrence of any type of cancer. All cases were identified from 2007 to 2010 in 15 of 16 Montreal hospitals that treat breast cancer. | Random sample from the universal Provincial Voter Registration List, approximately frequency-matched to cases on age (5-year bins) and living in Montreal. |
| NBCS* | Norwegian Breast Cancer Study [[26](#_ENREF_26)] | Norway | Incidence cases from three different hospitals: Ullevål Univ. Hospital 1990-94, Norwegian Radium Hospital 1975-1986 and 1995-1998, Haukeland Univ. Hospital 1992-2001. | Women residing in Tromsø and Bergen who attended the Norwegian Breast Cancer Screening Program. |
| OBCS | Oulu Breast Cancer Study [[27](#_ENREF_27)] | Finland | Consecutive incident cases diagnosed at the Oulu University Hospital between 2000-2004. | Female blood donors recruited in 2002 from the same geographical region in Northern Finland. |
| OFBCR^‡^ | Ontario Familial Breast Cancer Registry [[28](#_ENREF_28)] | Canada | Invasive cases aged 20-54 and a random sample aged 55-69 years identified from the Ontario Cancer Registry from 1996-1998. All those at high genetic risk were eligible; random samples of women not meeting these criteria were also asked to participate. During 2001-2005, enrolment was limited to minority and high-risk families. | Identified by calling randomly selected residential telephone numbers in the same geographical region from 1998-2001; frequency matched to cases by age in 5 year categories. |
| ORIGO | Leiden University Medical Centre Breast Cancer Study [[29](#_ENREF_29),[30](#_ENREF_30)] | Netherlands | Consecutive case patients diagnosed 1996–2006 in 2 hospitals in South–West Netherlands (Leiden & Rotterdam). No selection for family history; Rotterdam case patients selected for diagnosis aged <70. Case patients with in situ carcinomas eligible. | (1) Blood bank healthy donors from Southwest Netherlands recruited in 1996, 2000 or 2007; (2) People who married a person who was part of a family with high breast cancer risk (BRCA1/2/X). From the Southwest of the Netherlands, recruited 1990–1996; (3) Females tested at the local clinical genetics department for familial diseases, excluding familial cancer syndromes (no mutation found in gene(s) related to the disease being tested), recruited 1995–2007. |
| PBCS | NCI Polish Breast Cancer Study [[31](#_ENREF_31)] | Poland | Incident cases identified through a rapid identification system in participating hospitals covering ~ 90% of all eligible cases, and cancer registries in Warsaw and Łódź covering 100% of all eligible cases (2000-2003). | Randomly selected from population lists of all residents of Poland from 2000-2003, stratified and frequency matched to cases on city and age in 5-year categories. |
| pKARMA | Karolinska Mammography Project for Risk Prediction of Breast Cancer [[25](#_ENREF_25)] | Sweden | Incident cases from Jan 2001 – Dec 2008 from the Stockholm/Gotland area. Identified through the Stockholm breast cancer registry. | Unmatched participants of the KARMA mammography screening study recruited between 2010 and 2011 from Southern Sweden and Stockholm. |
| RBCS | Rotterdam Breast Cancer Study [[32](#_ENREF_32)] | Netherlands | Familial breast cancer patients selected from the clinical genetics center at Erasmus Medical Center between 1994-2005. | Spouses or mutation-negative siblings of heterozygous Cystic Fibrosis mutation carriers selected from the clinical genetics centre at Erasmus Medical Center between 1996-2006. |
| SASBAC | Singapore and Sweden Breast Cancer Study [[33](#_ENREF_33)] | Sweden | Women diagnosed in Sweden aged 50-74 in 1993-1995. | Population-based controls frequency matched by age to the cases. |
| SBCS | Sheffield Breast Cancer Study [[34](#_ENREF_34)] | U.K. | Women with breast cancer recruited in 1998-2005 at surgical outpatient clinics at the Royal Hallamshire Hospital, Sheffield. | Unselected women attending the Sheffield Mammography Screening Service in 2000-2004 with no evidence of a breast lesion. |
| SEARCH | Studies of Epidemiology and Risk Factors in Cancer Heredity [[35](#_ENREF_35),[36](#_ENREF_36)] | U.K. | Identified through the Eastern Cancer Registration and Information Centre: (i) prevalent cases; diagnosed 1991-1996; under 55 years of age at diagnosis; recruited 1996-2002 (ii) incident cases; diagnosed since 1996; under 70 years of age at diagnosis; recruited 1996-present. | (a) Women from the same geographic region selected from the EPIC-Norfolk cohort study, 1992-1994 (b) women attending GP practices, frequency matched to cases by age and geographic region (2003-2010) (c) women attending for breast screening as part of the NHSBSP participating in the Sisters in Breast Screening (SIBS) study |
| SKKDKFZ* | Städtisches Klinikum Karlsruhe Deutsches Krebsforschungszentrum Study [[37](#_ENREF_37)] | Germany | Women diagnosed with primary *in situ* or invasive breast cancer at the Städtisches Klinikum Karlsruhe from March 1993 to July 2005. Cases were 21-93 years of age. | Controls for triple negative cases were from an unselected series of unaffected women from the same geographical region. |
| SZBCS | Szczecin Breast Cancer Study [[38](#_ENREF_38)] | Poland | Prospectively ascertained cases of invasive breast cancer patients diagnosed at the Regional Oncology Hospital (2002-2003 and 2006-2007) or the University Hospital (2002-2007), both in Szczecin, West Pomerania, Poland. | Selected from a population-based study of the 1.3 million inhabitants of West Pomerania (2003-2004); matched to cases for year of birth, sex and region. |
| TNBCC* | Triple Negative Breast Cancer Consortium Study (Stevens *et al*) | Various | Triple negative invasive breast cancer cases from multiple countries | Women free of breast cancer from the same geographic regions as cases |
| UKBGS | Breakthrough Generations Study [[39](#_ENREF_39)] | U.K. | Cohort members who developed breast cancer or in situ breast cancer after entry into the Breakthrough Generations Study (cohort of >100,000 women followed up for breast cancer, recruited from the UK during 2003-2010). | Women who had not had breast cancer or in situ breast cancer selected by 1:1 matching to cases on date of birth, year of entry in to the study (2003-2010),  source of recruitment, availability of blood sample and ethnicity. |

| ACP | Asian Cancer Project | Thailand | Cases from oncology centres in Thailand that underwent biopsy and had been pathologically diagnosed as having breast cancer. | Hospital based controls are women who were admitted to the same hospital as the cases with diseases not related to cancer or metabolic  syndromes such as diabetes, heart diseases or conditions related to gynaecology. |
| --- | --- | --- | --- | --- |
| HERPACC | Hospital-based  Epidemiologic Research  Program at Aichi Cancer  Center [[40](#_ENREF_40)] | Japan | Incident breast cancer cases who first visited Aichi  Cancer Center between 2001 and 2005 and were diagnosed within 1 year from the first visit. No previous history of any type of cancer. | Controls were selected from pool of non-cancer patients who firstly visited Aichi Cancer Center between 2001-2005. Non-cancer status is defined as "having no positive finding on any of clinical/laboratory/graphical examination within 1 year from their fist visit. No previous history of cancer is allowed. |
| LAABC | Los Angeles County  Asian-American Breast  Cancer Case-Control [[41](#_ENREF_41)] | USA | Incident cases recruited from 1995-2007 and  identified from SEER cancer registries in Los  Angeles County. Grouped by self-reported ethnicity | Controls were recruited during 1995-  2009 and selected from the same neighbourhood as where cancer cases resided at the time of diagnosis. Controls were frequency matched to the cases on specific Asian ethnicities and 5-year age groups. |
| MYBRCA | Malaysian Breast Cancer  Genetic Study [[42](#_ENREF_42)] | Malaysia | Breast cancer cases identified at the Breast Cancer  Clinic in University Malaya Medical Centre Jan  2001-July 2010; cases are a mixture of prevalent  and incident cases | Controls are cancer-free individuals (21-70 years) with no breast or ovarian cancer in first degree  relatives, randomly selected from women attending same hospital. |
| SBCGS | Shanghai Breast Cancer  Genetic Study [[43](#_ENREF_43)] | China | Newly-diagnosed breast cancer cases recruited from 1996 -2009. Cases were identified mostly from the Shanghai Cancer Registry. Some cases  were identified from the Shanghai Women’s  Health Study. | Community controls randomly selected from the general population using the resident registry or from cancer-free cohort members in the Shanghai Women’s Health Study. The controls were recruited from the same geographical region as cases during 1996-2009. |
| SEBCS | Seoul Breast Cancer  Study [[44](#_ENREF_44)] | South Korea | Consecutive, incident, cases from 2 hospitals in  Seoul recruited between 2001-2005. | Women from same catchment area and participating in annual health check-up (2001-2005). |

| SGBCC | Singapore Breast Cancer  Cohort | Singapore | Living breast cancer patients diagnosed with  primary in-situ or invasive breast cancer at  National University Hospital. Cases are a mixture  of prevalent and incident cases. | All community-dwelling individuals who are Singaporeans or Singaporean Permanent Residents, 21 years and older. Exclusion criteria were a medical history of cancer,  acute myocardial infarction or stroke, or major psychiatric morbidity including schizophrenia, psychotic depression, and advanced Alzheinmer's Disease. |
| --- | --- | --- | --- | --- |
| TBCS | IARC-Thai Breast  Cancer [[45](#_ENREF_45)] | Thailand | Incident cases diagnosed at the National Cancer  Institute (NCI) in Bangkok and Khon Kaen Hospital during 2002-2004. | Randomly selected women visiting  hospital patients with diseases other  than breast or ovarian cancer at NCI  Bangkok and Khon Kaen Hospital  during 2002- 2004). |
| TWBCS | Taiwanese Breast Cancer Study [[46](#_ENREF_46)] | Taiwan | Incident cases diagnosed & treated at 2 major teaching hospitals in Taiwan between 2002-2005. | Randomly selected women attending  a health examination at same  hospitals between 2002-2005. |
| SCCS | Southern Community  Cohort Study [[47](#_ENREF_47)] | USA | Incident cases (N=222) from 2002-2009 identified from 12 state cancer registries in southeastern US.  Prevalent cases (N=493) identified by self-reported  history of breast cancer reported on cohort enrollment survey. | Controls for the incident cases were individually matched on age (+/- 2 years), self-reported race, menopausal status, enrollment site, and date of sample collection (+/- 6months). Controls for the prevalent cases were frequency matched on age (+/- 1 year) and enrollment site. |

| NBHS | Nashville Breast Health  Study [[43](#_ENREF_43)] | USA | Through a rapid case-ascertainment system, we  identified newly-diagnosed breast cancer cases  through the Tennessee State Cancer Registry and  five major hospitals in the city that provide  medical care for breast cancer patients. Eligible  cases were women diagnosed with invasive breast  cancer or ductal carcinoma in situ, who were  between the ages of 25 and 75, had no prior  history of cancer other than non-melanoma skin  cancer, had a resident telephone, spoke English,  and who were able to provide consent to the  study. Recruitment period was from 2001 to 2011.  The recruitment for European Americans ended in  2008. | Controls were identified via random digit dialing (RDD) of households in the same geographic area as cases during 2001-2011. Eligibility criteria for controls were the same as cases with the exception that controls did  not have a prior cancer diagnosis other than simple skin cancer. Controls were frequency matched to cases on 5-year age group, race, and country of residence. |
| --- | --- | --- | --- | --- |

BCAC, Breast Cancer Association Consortium; COGS, Collaborative Oncological Gene-environment Study; * CTS, NBCS and SKKDKFZ are studies in BCAC but were genotyped as part of the triple negative consortium (TNBCC). Part of GENICA was also genotyped as part of TNBCC.

^†^Australian site of the Breast Cancer Family Registry; ^‡^Ontario site of the Breast Cancer Family Registry

**References:**

1. Dite GS, Jenkins MA, Southey MC, Hocking JS, Giles GG, et al. (2003) Familial risks, early-onset breast cancer, and BRCA1 and BRCA2 germline mutations. J Natl Cancer Inst 95: 448-457.

2. Schmidt MK, Tollenaar RA, de Kemp SR, Broeks A, Cornelisse CJ, et al. (2007) Breast cancer survival and tumor characteristics in premenopausal women carrying the CHEK2*1100delC germline mutation. J Clin Oncol 25: 64-69.

3. Schrauder M, Frank S, Strissel PL, Lux MP, Bani MR, et al. (2008) Single nucleotide polymorphism D1853N of the ATM gene may alter the risk for breast cancer. J Cancer Res Clin Oncol 134: 873-882.

4. Fletcher O, Johnson N, Palles C, dos Santos Silva I, McCormack V, et al. (2006) Inconsistent association between the STK15 F31I genetic polymorphism and breast cancer risk. J Natl Cancer Inst 98: 1014-1018.

5. Colleran G, McInerney N, Rowan A, Barclay E, Jones AM, et al. (2010) The TGFBR1*6A/9A polymorphism is not associated with differential risk of breast cancer. Breast Cancer Res Treat 119: 437-442.

6. Yang R, Dick M, Marme F, Schneeweiss A, Langheinz A, et al. (2011) Genetic variants within miR-126 and miR-335 are not associated with breast cancer risk. Breast Cancer Res Treat 127: 549-554.

7. Villeneuve S, Fevotte J, Anger A, Truong T, Lamkarkach F, et al. (2011) Breast cancer risk by occupation and industry: analysis of the CECILE study, a population-based case-control study in France. Am J Ind Med 54: 499-509.

8. Weischer M, Bojesen SE, Tybjaerg-Hansen A, Axelsson CK, Nordestgaard BG (2007) Increased risk of breast cancer associated with CHEK2*1100delC. J Clin Oncol 25: 57-63.

9. Milne RL, Ribas G, Gonzalez-Neira A, Fagerholm R, Salas A, et al. (2006) ERCC4 associated with breast cancer risk: a two-stage case-control study using high-throughput genotyping. Cancer Res 66: 9420-9427.

10. Bernstein L, Allen M, Anton-Culver H, Deapen D, Horn-Ross PL, et al. (2002) High breast cancer incidence rates among California teachers: results from the California Teachers Study (United States). Cancer Causes Control 13: 625-635.

11. Widschwendter M, Apostolidou S, Raum E, Rothenbacher D, Fiegl H, et al. (2008) Epigenotyping in peripheral blood cell DNA and breast cancer risk: a proof of principle study. PLoS One 3: e2656.

12. Pesch B, Ko Y, Brauch H, Hamann U, Harth V, et al. (2005) Factors modifying the association between hormone-replacement therapy and breast cancer risk. Eur J Epidemiol 20: 699-711.

13. Justenhoven C, Pierl CB, Haas S, Fischer HP, Baisch C, et al. (2008) The CYP1B1_1358_GG genotype is associated with estrogen receptor-negative breast cancer. Breast Cancer Res Treat 111: 171-177.

14. Heikkinen T, Karkkainen H, Aaltonen K, Milne RL, Heikkila P, et al. (2009) The breast cancer susceptibility mutation PALB2 1592delT is associated with an aggressive tumor phenotype. Clin Cancer Res 15: 3214-3222.

15. Bogdanova NV, Antonenkova NN, Rogov YI, Karstens JH, Hillemanns P, et al. (2010) High frequency and allele-specific differences of BRCA1 founder mutations in breast cancer and ovarian cancer patients from Belarus. Clin Genet 78: 364-372.

16. Margolin S, Werelius B, Fornander T, Lindblom A (2004) BRCA1 mutations in a population-based study of breast cancer in Stockholm County. Genet Test 8: 127-132.

17. Hartikainen JM, Tuhkanen H, Kataja V, Dunning AM, Antoniou A, et al. (2005) An autosome-wide scan for linkage disequilibrium-based association in sporadic breast cancer cases in eastern Finland: three candidate regions found. Cancer Epidemiol Biomarkers Prev 14: 75-80.

18. Beesley J, Jordan SJ, Spurdle AB, Song H, Ramus SJ, et al. (2007) Association between single-nucleotide polymorphisms in hormone metabolism and DNA repair genes and epithelial ovarian cancer: results from two Australian studies and an additional validation set. Cancer Epidemiol Biomarkers Prev 16: 2557-2565.

19. De Maeyer L, Van Limbergen E, De Nys K, Moerman P, Pochet N, et al. (2008) Does estrogen receptor negative/progesterone receptor positive breast carcinoma exist? J Clin Oncol 26: 335-336; author reply 336-338.

20. Flesch-Janys D, Slanger T, Mutschelknauss E, Kropp S, Obi N, et al. (2008) Risk of different histological types of postmenopausal breast cancer by type and regimen of menopausal hormone therapy. Int J Cancer 123: 933-941.

21. Catucci I, Verderio P, Pizzamiglio S, Manoukian S, Peissel B, et al. (2009) SNPs in ultraconserved elements and familial breast cancer risk. Carcinogenesis 30: 544-545; author reply 546.

22. Olson JE, Ma CX, Pelleymounter LL, Schaid DJ, Pankratz VS, et al. (2007) A comprehensive examination of CYP19 variation and breast density. Cancer Epidemiol Biomarkers Prev 16: 623-625.

23. Giles GG, English DR (2002) The Melbourne Collaborative Cohort Study. IARC Sci Publ 156: 69-70.

24. Kolonel LN, Altshuler D, Henderson BE (2004) The multiethnic cohort study: exploring genes, lifestyle and cancer risk. Nat Rev Cancer 4: 519-527.

25. Michailidou K, Hall P, Gonzalez-Neira A, Ghoussaini M, Dennis J, et al. (2013) Large-scale genotyping identifies 41 new loci associated with breast cancer risk. Nat Genet 45: 353-361.

26. Nordgard SH, Johansen FE, Alnaes GI, Bucher E, Syvanen AC, et al. (2008) Genome-wide analysis identifies 16q deletion associated with survival, molecular subtypes, mRNA expression, and germline haplotypes in breast cancer patients. Genes Chromosomes Cancer 47: 680-696.

27. Erkko H, Xia B, Nikkila J, Schleutker J, Syrjakoski K, et al. (2007) A recurrent mutation in PALB2 in Finnish cancer families. Nature 446: 316-319.

28. John EM, Hopper JL, Beck JC, Knight JA, Neuhausen SL, et al. (2004) The Breast Cancer Family Registry: an infrastructure for cooperative multinational, interdisciplinary and translational studies of the genetic epidemiology of breast cancer. Breast Cancer Res 6: R375-389.

29. de Bock GH, Schutte M, Krol-Warmerdam EM, Seynaeve C, Blom J, et al. (2004) Tumour characteristics and prognosis of breast cancer patients carrying the germline CHEK2*1100delC variant. J Med Genet 41: 731-735.

30. Huijts PE, Vreeswijk MP, Kroeze-Jansema KH, Jacobi CE, Seynaeve C, et al. (2007) Clinical correlates of low-risk variants in FGFR2, TNRC9, MAP3K1, LSP1 and 8q24 in a Dutch cohort of incident breast cancer cases. Breast Cancer Res 9: R78.

31. Garcia-Closas M, Brinton LA, Lissowska J, Chatterjee N, Peplonska B, et al. (2006) Established breast cancer risk factors by clinically important tumour characteristics. Br J Cancer 95: 123-129.

32. Hollestelle A, Pelletier C, Hooning M, Crepin E, Schutte M, et al. (2011) Prevalence of the variant allele rs61764370 T>G in the 3'UTR of KRAS among Dutch BRCA1, BRCA2 and non-BRCA1/BRCA2 breast cancer families. Breast Cancer Res Treat 128: 79-84.

33. Wedren S, Lovmar L, Humphreys K, Magnusson C, Melhus H, et al. (2004) Oestrogen receptor alpha gene haplotype and postmenopausal breast cancer risk: a case control study. Breast Cancer Res 6: R437-449.

34. MacPherson G, Healey CS, Teare MD, Balasubramanian SP, Reed MW, et al. (2004) Association of a common variant of the CASP8 gene with reduced risk of breast cancer. J Natl Cancer Inst 96: 1866-1869.

35. Easton DF, Pooley KA, Dunning AM, Pharoah PD, Thompson D, et al. (2007) Genome-wide association study identifies novel breast cancer susceptibility loci. Nature 447: 1087-1093.

36. Lesueur F, Pharoah PD, Laing S, Ahmed S, Jordan C, et al. (2005) Allelic association of the human homologue of the mouse modifier Ptprj with breast cancer. Hum Mol Genet 14: 2349-2356.

37. Rashid MU, Jakubowska A, Justenhoven C, Harth V, Pesch B, et al. (2005) German populations with infrequent CHEK2*1100delC and minor associations with early-onset and familial breast cancer. Eur J Cancer 41: 2896-2903.

38. Jakubowska A, Jaworska K, Cybulski C, Janicka A, Szymanska-Pasternak J, et al. (2009) Do BRCA1 modifiers also affect the risk of breast cancer in non-carriers? Eur J Cancer 45: 837-842.

39. Swerdlow AJ, Jones ME, Schoemaker MJ, Hemming J, Thomas D, et al. (2011) The Breakthrough Generations Study: design of a long-term UK cohort study to investigate breast cancer aetiology. Br J Cancer 105: 911-917.

40. Kawase T, Matsuo K, Suzuki T, Hiraki A, Watanabe M, et al. (2009) FGFR2 intronic polymorphisms interact with reproductive risk factors of breast cancer: results of a case control study in Japan. Int J Cancer 125: 1946-1952.

41. Wu AH, Yu MC, Tseng CC, Stanczyk FZ, Pike MC (2009) Dietary patterns and breast cancer risk in Asian American women. Am J Clin Nutr 89: 1145-1154.

42. Thirthagiri E, Lee SY, Kang P, Lee DS, Toh GT, et al. (2008) Evaluation of BRCA1 and BRCA2 mutations and risk-prediction models in a typical Asian country (Malaysia) with a relatively low incidence of breast cancer. Breast Cancer Res 10: R59.

43. Zheng W, Long J, Gao YT, Li C, Zheng Y, et al. (2009) Genome-wide association study identifies a new breast cancer susceptibility locus at 6q25.1. Nat Genet 41: 324-328.

44. Han S, Lee KM, Choi JY, Park SK, Lee JY, et al. (2008) CASP8 polymorphisms, estrogen and progesterone receptor status, and breast cancer risk. Breast Cancer Res Treat 110: 387-393.

45. Sangrajrang S, Schmezer P, Burkholder I, Waas P, Boffetta P, et al. (2008) Polymorphisms in three base excision repair genes and breast cancer risk in Thai women. Breast Cancer Res Treat 111: 279-288.

46. Ding SL, Yu JC, Chen ST, Hsu GC, Kuo SJ, et al. (2009) Genetic variants of BLM interact with RAD51 to increase breast cancer susceptibility. Carcinogenesis 30: 43-49.

47. Signorello LB, Hargreaves MK, Blot WJ (2010) The Southern Community Cohort Study: investigating health disparities. J Health Care Poor Underserved 21: 26-37.
